# Navigating Classical and Nonclassical Realms of Nucleation and Crystallization

**DOI:** 10.1002/smll.202511608

**Published:** 2026-08-17

**Authors:** Stephan E. Wolf, Peter G. Vekilov

**Affiliations:** ^1^ Department of Materials Science and Engineering Institute for Glass and Ceramics Friedrich‐Alexander University Erlangen‐Nürnberg (FAU) Erlangen Germany; ^2^ William A. Brookshire Department of Chemical and Biomolecular Engineering University of Houston Houston Texas USA

**Keywords:** crystal growth, crystallization, mesocrystals, nonclassical, nucleation, terminology

## Abstract

Our understanding of the formation and growth of solids from solution has shaped science for centuries. The classical nucleation and crystal growth theories provided a foundational framework, despite their necessary simplifications. Recent nonclassical concepts have challenged this canon and refined the general models of the molecular and mesoscale processes underlying solid‐state genesis. This shift of paradigms triggered novel terminology, often coined *ad hoc* to describe unexpected observations, but also created ambiguity across disciplinary boundaries. Here, we review current terminology, summarize the perceived classical canon, clarify ambiguous concepts, correct recurring misconceptions, and highlight terms that require further specification. We draw connections between colloidal, molecular, and mineral systems to develop generalizing concepts, while emphasizing where analogous phenomenology arises from distinct mechanistic or stabilizing conditions. We further identify the Szilard postulate—that crystallization proceeds by addition of individual ions or molecules—as a key criterion for distinguishing classical from nonclassical nucleation and growth mechanisms. By clarifying terminology and conceptual boundaries, this review provides a reference framework and a field guide for current debates on classical and nonclassical nucleation and crystallization.

## Graphical abstract

Classical nucleation and growth theories have long been foundational, yet recent nonclassical concepts have challenged and refined our understanding of solid‐state genesis. This paradigm shift introduced new, often ambiguous vocabulary. Here, we clarify key concepts, correct common misconceptions, and provide a terminological field guide across systems, emphasizing the Szilard postulate as a discriminator between classical and nonclassical mechanisms.

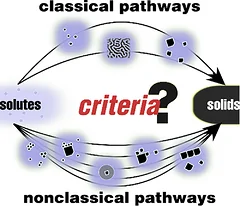

## 1 Introduction: Centennial Theories in Modern Research

Nucleation and crystallization in solutions are fundamental processes essential for myriad biological, geological, and synthetic phenomena. Regulation of nucleation and crystal growth enables the design of crucial features of the solid products, including polymorph structure, crystal morphology, defect density, and particle size distribution. Crystals constitute the primary form of solid‐state materials in the geo‐ and biospheres, rendering the understanding of crystal genesis crucial in life sciences and geosciences. In fact, biomineralizing organisms exemplify the impressive level of precision that is achievable in mineralization control. Various “simple” organisms transform ordinary, non‐functional minerals like chalk, rust, or apatite into vital organs used for sensing, locomotion, defense, or assault. These biosynthetic materials are unparalleled in their multifunctional design, sustainability, and damage resilience. Today, they serve as blueprints for the development of new classes of bioinspired materials [1, 2].

Untangling the underlying processes of phase separation and providing precise models with sufficient predictive power remains an interdisciplinary challenge. It thus comes with no surprise that research in phase separation and crystallization has a long history, built by generations of scholars from early crystallography to modern materials science. Today, we stand on their shoulders, equipped with ever‐advancing characterization technologies. The advent of high‐resolution in situ techniques unraveled unforeseen details concerning nucleation, crystallization, and solution processes in the pre‐nucleation stage. These findings sparked conceptual changes, referred to as nonclassical nucleation and crystallization. They challenge established views on phase separation and crystallization.

These paradigmatic changes brought in new terminology, which was often derived *ad hoc* while our understanding is still incomplete. Different disciplines are involved, which adds to potential confusion of the employed terms. In this review, we clarify key terminological issues, highlight ambiguous notions, and provide background for currently employed concepts. We aim to contribute to a harmonized vocabulary and to offer a field guide for scholars entering this dynamic research area. Section 2 summarizes the accepted classical canon of particle nucleation and crystal growth. Section 3 contrasts this canon with recent “nonclassical” works and models that escape its assumptions. Section 4 provides definitions, resolves common misinterpretations, and highlights terminological ambiguities arising from the interdisciplinary character of the field. Further, we draw connections between behavioral similarities of nonclassical inorganic systems that feature supra‐ionic species with molecular and colloidal systems, while emphasizing where analogous phenomenology arises from distinct mechanistic or stabilizing conditions. Section 5 concludes with an outlook on the broader debate between classical and nonclassical schools of thought.

Throughout this review, we provide *inline definitions* highlighted in italics; this review mainly adheres to terminological definitions put forward by international committees, e.g., the international union of pure and applied chemistry (IUPAC, as given in the compendium of chemical terminology, informally also known as the gold book [3]) or the international union of crystallography (IUCr).

## 2 Canonical Concepts of Classical Nucleation and Crystal Growth

### 2.1 Phases and Solute Species

The definition of a *phase* starts with the distinction between homogeneous and heterogeneous systems. In a *homogeneous system*, all properties are uniform or change smoothly; the extension of the definition to smooth property variations accounts, for instance, for a fluid flowing in a pipe driven by a pressure gradient, which, nonetheless, is still homogeneous. A *heterogeneous system* is one in which some of the properties undergo abrupt changes at some locations. From here, a *phase* is a homogeneous part of a heterogeneous system [4]. The locations at which at least one of the parameters of a system changes abruptly represent the *phase boundary*. Thus, a homogeneous system is the same as a single‐phase system, and a multiphase system is equivalent to a heterogeneous system. A phase does not need to be continuous and may consist of numerous domains. Indeed, a population of crystals (see Glossary) constitutes a single phase as long as their compositions and lattice structures, the determinants of their properties, are identical. The phase boundary is often idealized as an infinitesimally thin boundary layer, denoted as the Gibbs dividing plane or an ideal interface. Guggenheim and others extended the conceptualization of a two‐dimensional interface to a more realistic three‐dimensional *interphase* with graded properties and/or compositions.

A *phase diagram* is a graphic representation of the regions in the space of select physical parameters (e.g., temperature *T*, pressure *p*, volume *V*, composition *x*) in which the phases in a system exist. The phase diagram may reflect the regions of existence of *stable phases*, i.e., those whose formation minimizes the free energy of a system. *Metastable phases*, whose free energy is higher than that of another phase possible at the same conditions, but whose transformation to that stable phase is hindered by a thermodynamic barrier, are often included in phase diagrams. Often, regions in a phase diagram are assigned to *kinetically stabilized phases*, whose transformation to a more stable phase is delayed by non‐thermodynamic factors, such as viscosity. Often, the factors that stabilize a phase are not understood and, in some systems, the distinction between metastable and kinetically stabilized phases is diffuse.

Phase diagrams can be two‐dimensional. For instance, a (*p, T*) phase diagram of a pure substance identifies the combinations of pressure and temperature for which a certain phase, i.e., liquid, solid, or gas, is stable. *Phase lines* designate the (*p, T*) combinations for which two phases, such as liquid and a gas, are in equilibrium. Crossing a phase line by, for instance, increasing the temperature, indicates the transformation from solid to liquid. Importantly, the phase lines between stable phases are infinitely thin, i.e., an infinitesimal variation of *T* transforms all molecules of a solid to liquid. This postulate may appear counterintuitive until one realizes that thermodynamic equilibrium assumes that infinite time is available. Three phases may be in equilibrium at a *triple point*. Water has a triple point at 273.16 K and 611.73 Pa; numerous other triple points are present in the phase diagram of water at high pressures and low temperatures, indicating the existence of up to 20 known forms of ice.

Many properties and behaviors of pure substances, such as condensation upon compression of a gas, are better understood with the help of (*p, V*) phase diagrams. A complete description of the phase equilibria of a pure substance requires three‐dimensional phase diagrams (*p, V, T*).

**FIGURE 1:**
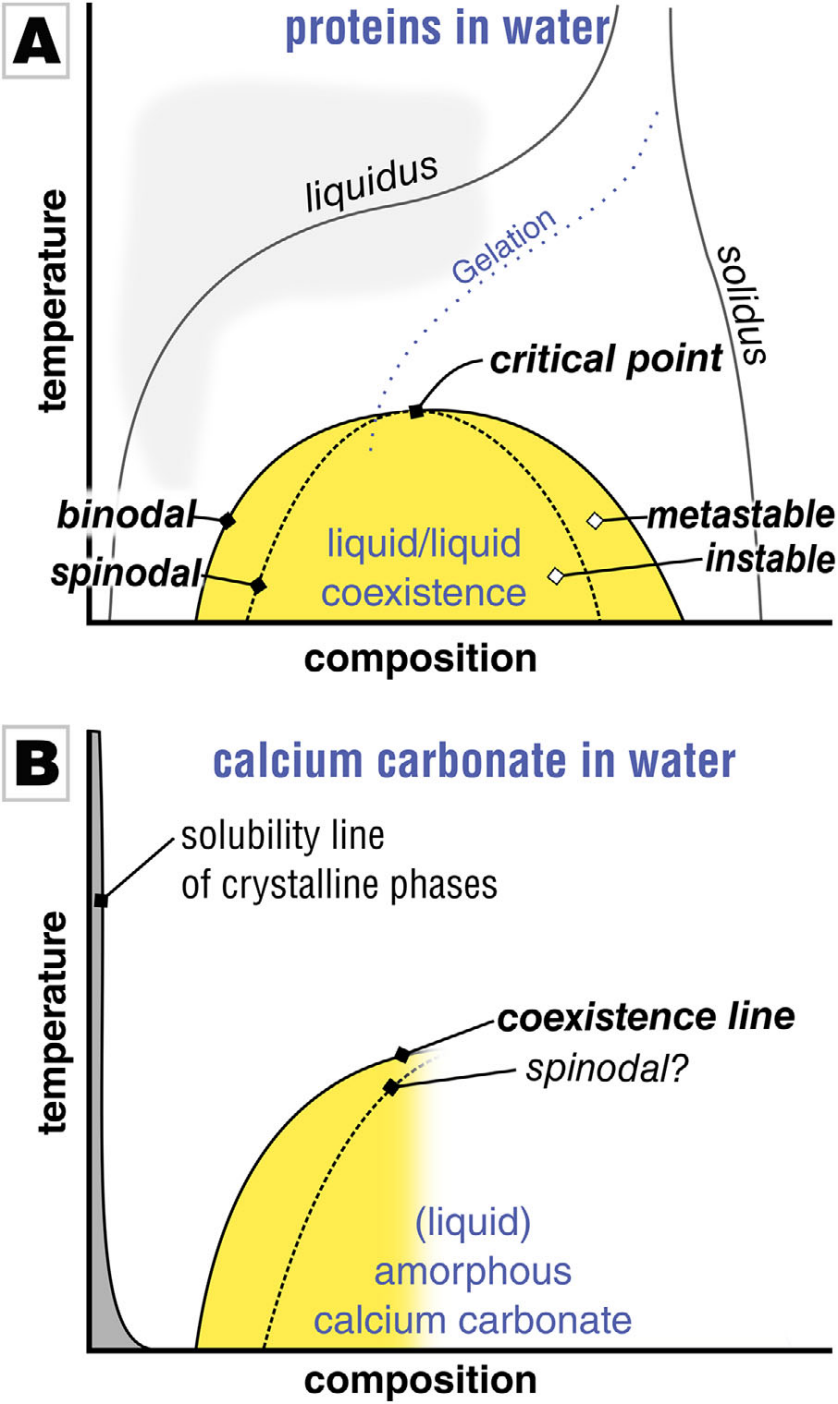
Two examples of phase diagrams. (A) A phase diagram typical for a protein solution. The area shaded in gray denotes those regions in which metastable protein aggregates/associates have been reported. (B) A conceptual phase diagram of calcium carbonate solutions. The crystalline phases are all sparingly soluble and show retrograde behavior; that is, their solubility increases with decreasing temperature. At higher supersaturation, amorphous calcium carbonate forms [6, 7]; liquid (‐like) calcium carbonate phases have also been evidenced [8, 9, 10], and spinodal demixing is discussed as a potential route to the latter [11]. Adapted with permission from (A) Vekilov 2009 [12], Ann. NY Acad. Sci., Wiley. (B) Scheme adapted from Wallace et al. [11]. 2013, AAAS, and enhanced with experimental findings from Zou et al. [7].

A system may contain numerous compounds with distinct chemical identities, which are referred to as *components*. A *solute* is a minority constituent of a solution, dissolved in a *solvent*, which represents the majority component. Solutions may be solid, for instance metal alloys, or liquid, such as seawater, or most physiological fluids. A solution may contain multiple solutes, such as alcohol, sugar, and many others in wine. Dissolved ions, such as in the case of dissolved CaCO_3_, may form supra‐ionic species such as ion pairs (see Glossary), and the speciation of the individual ions may change as a function, e.g., of pH (especially for salts of weak acids or bases, see the corresponding Hägg or Bjerrum plots) or temperature [5]. *Solution phase diagrams* are commonly presented in (*T, x*) coordinates (where *x* is the solute mole fraction), see examples given in Figure 1. In such phase diagrams, the line of coexistence between two stable liquids (i.e., liquid/liquid coexistence line) is referred to as *binodal*, and the lines designating the compositions of a liquid and solid in equilibrium with each other are called *liquidus* and *solidus*, respectively. Sections of the liquidus are sometimes called *solubility lines*.

The number of phases that a compound or solution may form is determined by the chemical properties of the components and is not limited by thermodynamics. The number of phases that can coexist, however, and how pressure and volume affect the regions of coexistence is governed by the *Gibbs phase rule*. This rule answers why we call the regions of coexistence of two phases a phase line and why the region of coexistence of three phases is limited to a unique combination of pressure and temperature, the triple point. The Gibbs phase rule relates the number of degrees of freedom in a system, *F*, i.e., the number of thermodynamic variables, which can be changed without losing or gaining a phase, with the number of components *C*, and the number of coexisting phases *P*, *F* = *C* − *P* + 2. According to the Gibbs phase rule, a pure substance existing in a unique phase has two degrees of freedom, i.e., we can change both *P* and *T* without generating a second phase. If, however, gas and liquid coexist, *P* = 2 and *F* = 1, implying that the pressure *P* must follow the variations in *T* lest we lose a phase, thus creating a phase line in the (*p, T*) plane. The phase rule shows that three phases can coexist at conditions without any degrees of freedom, i.e., only at the combination of pressure and temperature at the triple point, and that the coexistence of four phases of a pure substance is impossible. If *C* = 2 or more, we get that two phases may coexist in a region of the (*T, x*) phase diagram, in which both parameters may be independently varied.

**FIGURE 2:**
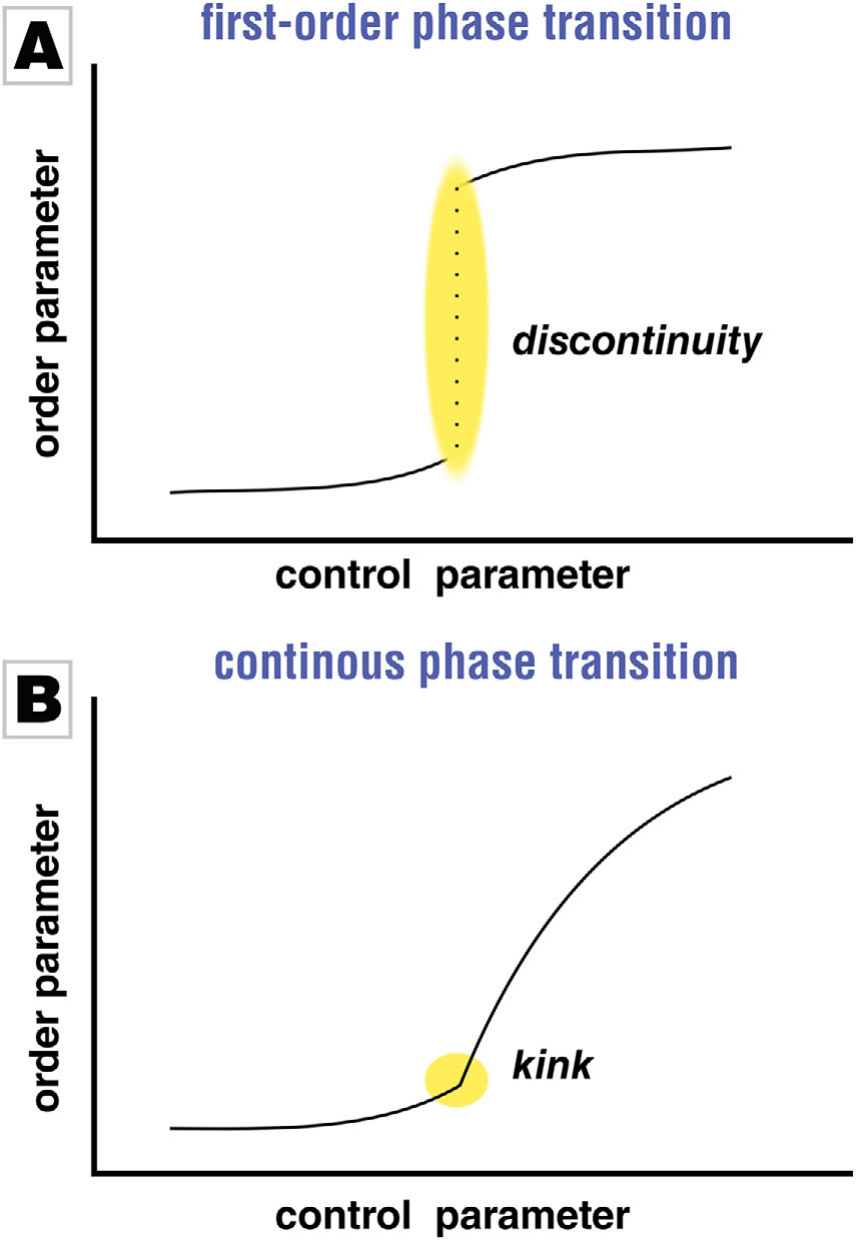
(A) First‐order transitions show discontinuities in a physical property (e.g., volume), the order parameter, upon slight changes of the control parameter (e.g., temperature). (B) Continuous or second‐order transitions show a kink instead of a jump in the order parameter. Adopted from Erdel [14].

The phase lines denote *first‐order phase transitions*, marking discontinuities in the first derivative of the free energy. For instance, a typical phase transition such as liquid to solid or vapor shows a discontinuity in (∂*G*/∂*P*)_*T*_ = *V*. In the Ehrenfest classification, the order of a phase transition equals the lowest derivative of the free energy that features discontinuities during the phase transition (Figure 2A) [4, 13]. In the 1970s, a simplified classification scheme replaced the earlier Ehrenfest classification [13]: first‐order phase transitions are characterized by latent heat in contrast to *second‐order (or continuous) transitions* (Figure 2B). Thus, glass transitions are second‐order transitions [4, 13].

### 2.2 Phase Separation Processes

***Phase separation***, the formation of one or more new phases from a parental phase [4], can occur via several processes and requires that the system is taken out of its phase region of stability by crossing phase lines into the regions of stability of other phases. While phase diagrams and their lines describe equilibrium states, *phase transitions* represent transformation from one equilibrium state to another equilibrium state. If the new phase forms slowly, a system may cross a phase line without immediate phase separation, thus becoming metastable with respect to the stable phase in the new phase regions. We will revisit this again in the context of the Ostwald‐Miers region, *vide infra*.

The current concept of two distinct phase separation mechanisms was put forward by Gibbs in 1876. He postulated that a multi‐component system only remains in the single‐phase regime if it resists two types of concentration fluctuations: (a) “large in degree but small in extent” and (b) “small in degree and large in extent” [15, 16]. The first scenario addresses *binodal phase separation* in metastable systems; the process of overcoming the barrier for the formation of a new phase is called *nucleation*. Gibbs attributed the barrier that prevents the old phase from immediately transforming into a new phase to the surface free energy of the interface between the first small domain of the new phase and evaluated the height of that barrier. The second case covers phase separation in unstable systems, for which the formation of a new phase does not encounter a barrier and is called *spinodal decomposition*.

***Classical nucleation theory*** (CNT) — put forth in the 1920s and 30s — covers Gibbs' first scenario, addressing the question at which point a localized concentration fluctuation is sufficiently “large in degree” to represent a new phase. Thus, in short, CNT assumes stochastic formation of new‐phase clusters, of which only those grow further into a stable particle of the new phase whose volume free energy outbalances its interface free energy. CNT modeled this considering the formation of a spherical liquid droplet with radius *r* in its supersaturated vapor. CNT was the first theoretical treatment of nucleation and was derived by Volmer, Becker, Döring, Zel'dovich, and Frenkel [17, 18, 19, 20]. Later, Turnbull and Fisher extended CNT to liquid/solid transitions (e.g., crystallization from solutions or melts) [21, 22].

Already in 1927, Szilard and Farkas introduced a crucial assumption that molecules from a supersaturated phase join a nucleus of the new phase individually, rather than as large groups or pre‐assembled clusters. They emphasized that dimers, trimers, and larger oligomers would have, according to Gibbs, higher free energy and that their concentrations would be too low to significantly contribute to the growth of the liquid droplet [23]. This **Szilard postulate** is a crucial assumption in CNT and a defining feature of all classical scenarios of crystal nucleation and growth.

If a single component is involved in a phase transformation, for instance, a molecular solute crystallizing from a multicomponent solution, the driving force for nucleation is the excess chemical potential of that component over that in the stable form, i.e., the crystal. In a supersaturated solution, the chemical potential Δμ for phase separation is given by

(1) $$\Delta \mu \approx k_{B}T\ln\sigma_{rel}\approx k_{B}T\left(\sigma_{rel}-1\right)$$

where σ_*rel*_ = *c* /*c_eq_* is the ratio of solute concentration *c* to the solubility *c_eq_*. The first approximate equality accounts for the neglect of the activity coefficients γ and γ of the solute in the supersaturated solution and at equilibrium, respectively, by assuming that γ = γ_*e*_. The second approximate equality only applies to low concentrations, for which the logarithmic function can be represented by the first two members of its Taylor series. Ionic crystals often form in solutions, in which the ratio of the concentration of the constituent ions is nonstoichiometric. In this case

(2) $$\Delta \mu =k_{B}T\ln\frac{IAP}{K_{SP}}=k_{B}T\ln S$$

with *IAP* as the ion activity product of an ionic solute, *K_SP_* the compound's solubility product, and *S* = *IAP* / *K_SP_* is the supersaturation. Both *IAP* and *K_SP_* are of similar form as the reaction quotient *Q_r_*, and they describe the present solution and the equilibrium state, respectively:

(3)

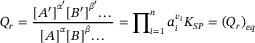

for a reaction of the type $\alpha \;A+\beta \;B+\cdots ⇌\alpha^{\prime }\;A^{\prime }+\beta^{\prime }B^{\prime }+\cdots$. The stoichiometric coefficients *v_i_* are set negative for the reactants (on the left side of the reaction equation) and positive for the products.

**FIGURE 3:**
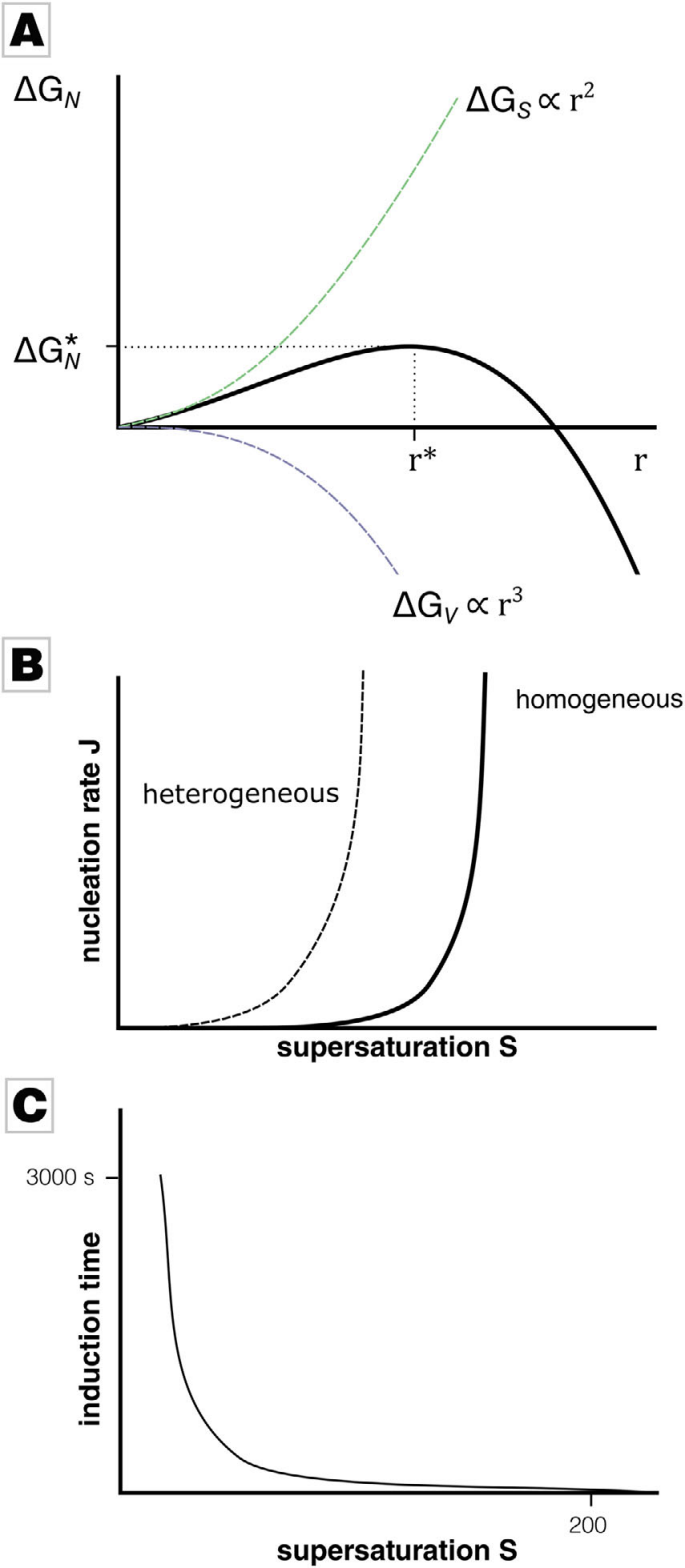
(A) Binary energy balance in classical nucleation theory. (B) Idealized schematic of nucleation rate *J* versus supersaturation *S*, contrasting heterogeneous and homogeneous nucleation in the same system at low to moderate *S*. Because the nucleation barrier is reduced under heterogeneous conditions, nucleation sets in at lower *S* than in the homogeneous case. The curves are qualitative; real systems may show additional dependence on experimental conditions and may deviate at high supersaturation owing to non‐constant kinetic prefactors, transport limitations, or alternative pathways such as spinodal decomposition. (C) Schematic induction time of calcium carbonate, on the basis of laboratory‐scale plant experiments [42].

A simplifying free energy balance with only two terms — the free energy of formation (also referred to as volume free energy) of the new phase Δ*G_V_* (*r*) and surface free energy of the nucleus Δ*G_S_* (*r*) — allowed Gibbs to identify the parameters that govern nucleation (Figure 3A). The most important of these parameters is the critical nucleus radius r*, which gives the size of a new phase domain after which its growth leads to free energy loss—according to the Second Law of thermodynamics, only processes that lead to a loss of the Gibbs free energy of the system are possible at constant temperature and pressure. A common misunderstanding is that a phase domain of size r* is stable and it has a 100% probability to grow. In fact, a critical nucleus with radius *r* = r* resting at the top of the free energy landscape is unstable, with 50% chance to dissociate and 50% chance to grow [24].

To understand the free energy variations in the vicinity of r*, we consider Δ*G_V_* (*r*) and Δ*G_S_* (*r*). For a supersaturated system, the bulk contribution to the free energy of formation of a new phase domain is negative Δ*G_V_* (*r*) = −*n*Δμ, where the negative sign accounts for the choice of the final state, the crystal, as a reference point for Δμ, in contrast to the thermodynamic convention used for Δ*G_V_*, in which the initial state is the reference. Assuming compact new phase domains, *n*∝*r* ^3^ and Δ*G_V_* scales with its volume, Δ*G_V_* (*r*)∝*r* ^3^ < 0. The surface free energy of the new phase domain α increases the free energy of the system, and its contribution scales with α, Δ*G_S_* (*r*)∝*r* ^2^ > 0.

(4) $$\begin{array}{ccc} \Delta G_{N}\left(r\right) & = & \Delta G_{V}\left(r\right)+\Delta G_{S}\left(r\right)=-\frac{4\pi r^{3}}{3\nu }\Delta \mu +4\pi r^{2}\alpha \\ & = & -\frac{4\pi r^{3}}{3\nu }k_{B}T\ln S+4\pi r^{2}\alpha \end{array}$$

(5) $$r^{*}=\frac{2\nu \alpha }{\Delta \mu }=\frac{2\nu \alpha }{k_{B}T\ln S}\;\mathrm{and}\;\Delta G_{N}^{*}\left(r=r^{*}\right)=\frac{16\pi \alpha^{3}v^{2}}{3k_{B}^{2}T^{2}{\left(\ln S\right)}^{2}}$$

where ν is the molecular volume in the new phase. The critical radius r* denotes the radius at which Δ*G_V_* outbalances Δ*G_S_*. Subcritical nuclei with *r* < r* are unstable and dissolve. Supercritical particles grow further, representing the new phase. Typical critical sizes of nuclei contain as few as 4 or 10 [25] up to 1000 [26] molecules and are thus on the nanometer scale. For the protein apoferritin, Yau and Vekilov directly observed a critical size of about 40 nm, comprising about 6 molecules, each 13 nm in diameter [27]. Indirect measurements indicate that the critical sizes for the proteins lysozyme, insulin, and sickle‐cell hemoglobin are similarly small [28, 29, 30, 31]. Observations of two‐dimensional crystalline layers are somewhat simpler, and measurements of the size of critical and near‐critical 2D nuclei at small sizes are consistent with the similarity between the thermodynamic analysis of two‐dimensional nucleation and that presented in Equations (4) and (5) [32, 33, 34].

Notably, nuclei smaller than about 10 molecules no longer comply with the main assumption of CNT that *r* changes smoothly. Detailed experiments have shown that the discontinuity is much more severe than the discreteness of the transition from 10 → 9 and can be 10 → 4 [28, 29, 30, 31]; the discontinuous transition represents one of the nonclassical scenarios discussed below.

The maximum of Δ*G_N_* (*r*) at the critical radius *r* = r* defines the *nucleation barrier* whose height $\Delta G_{N}^{*}$ governs the nucleation rate and thereby influences the kinetics and outcome of the phase separation process, including the particle size distribution. Volmer postulated that the nucleation rate *J_N_*, i.e., the number of new phase domains that appear in a unit volume of the supersaturated old phase per unit time, should follow an Arrhenius‐type expression [20]:

(6) $$J_{N}=J_{0}\exp\left(-\frac{\Delta G_{N}^{*}}{k_{B}T}\right)$$

According to Equations (5) and (6), the nucleation rate is extremely sensitive to temperature, supersaturation, or changes in interfacial energy (Figure 3B). Zeldovich proposed that *J*_0_ = *j_N_* · ϱ_*N*_ · *Z*, where *j_N_* is the frequency of attachment of growth units to the nucleus, ϱ_*N*_ as the number concentration of molecules, and Z is the Zeldovich factor, which accounts for the lower concentration of clusters of size r* in a system, in which nucleation proceeds at a steady rate, from an estimate that assumes, unphysically, that the system is in equilibrium [35].

Nucleation is thus thermally activated and, therefore, stochastic. This stochastic trait gives rise to a characteristic *induction time* $\tau \propto \exp(\gamma /T{\left(\log S\right)}^{2})$, a delay between crossing the binodal and nucleation or, in other words, a time‐lag between achieving supersaturation and the start of steady nucleation [19, 36], see Figure 3C. The experimentally measured nucleation delay time includes an additional contribution from the time needed for a particle to grow to detectable dimensions. This induction time is also the origin of the Ostwald–Miers region, which allows for supercooling.

CNT's conceptual accessibility and its qualitative predictive capabilities allowed it to survive the tests of time. Introduction of additional terms allows, for instance, straightforward handling of more complex phenomena, such as secondary nucleation [37] or heterogeneous nucleation [38, 39, 40]. In contrast to primary nucleation described by CNT (thus, in the absence of particles/crystals of the separating phase), *secondary nucleation* requires the presence of a parent crystal/particle that generates new crystallization seeds, e.g., by kinetic attrition and splintering [41]. This quasi‐autocatalytic mechanism bypasses the stochastic nature of classical primary nucleation and is thus extensively used to describe industrial processes. It must be distinguished from *heterogeneous nucleation*, the primary nucleation of a crystal on an interface. Heterogeneous nucleation can be described readily by introducing suitable corrective free energy terms into the original CNT equation: [38, 39, 40] Δ*G_V_* is corrected for a reduced volume of a nucleus formed on a substrate. Δ*G_S_* is expanded by terms that describe the change in interfacial energy, accounting for the interface changes between the nucleating phase (···_*n*_), the liquid phase (···_*l*_), and the nucleation substrate (···_*s*_) as a function of *r*. If the substrate/nucleus interfacial energy is lower than that of liquid / substrate (which it typically is, except for, e.g., highly hydrophilic interfaces), heterogeneous nucleation is kinetically favored over homogeneous nucleation. For secondary nucleation, α_*ns*_ becomes essentially zero and thus favorable over primary nucleation; moreover, most if not all of the critical nucleus volume is already provided.

(7) $$\Delta G_{N}^{het}\left(r\right)=\Delta G_{V}\left(r\right)+\Delta G_{S}\left(r\right)=-\frac{2\pi r^{3}}{3\nu }\Delta \mu +\pi r^{2}\left(2\alpha_{ln}+\alpha_{ns}-\alpha_{ls}\right)$$

**FIGURE 4:**
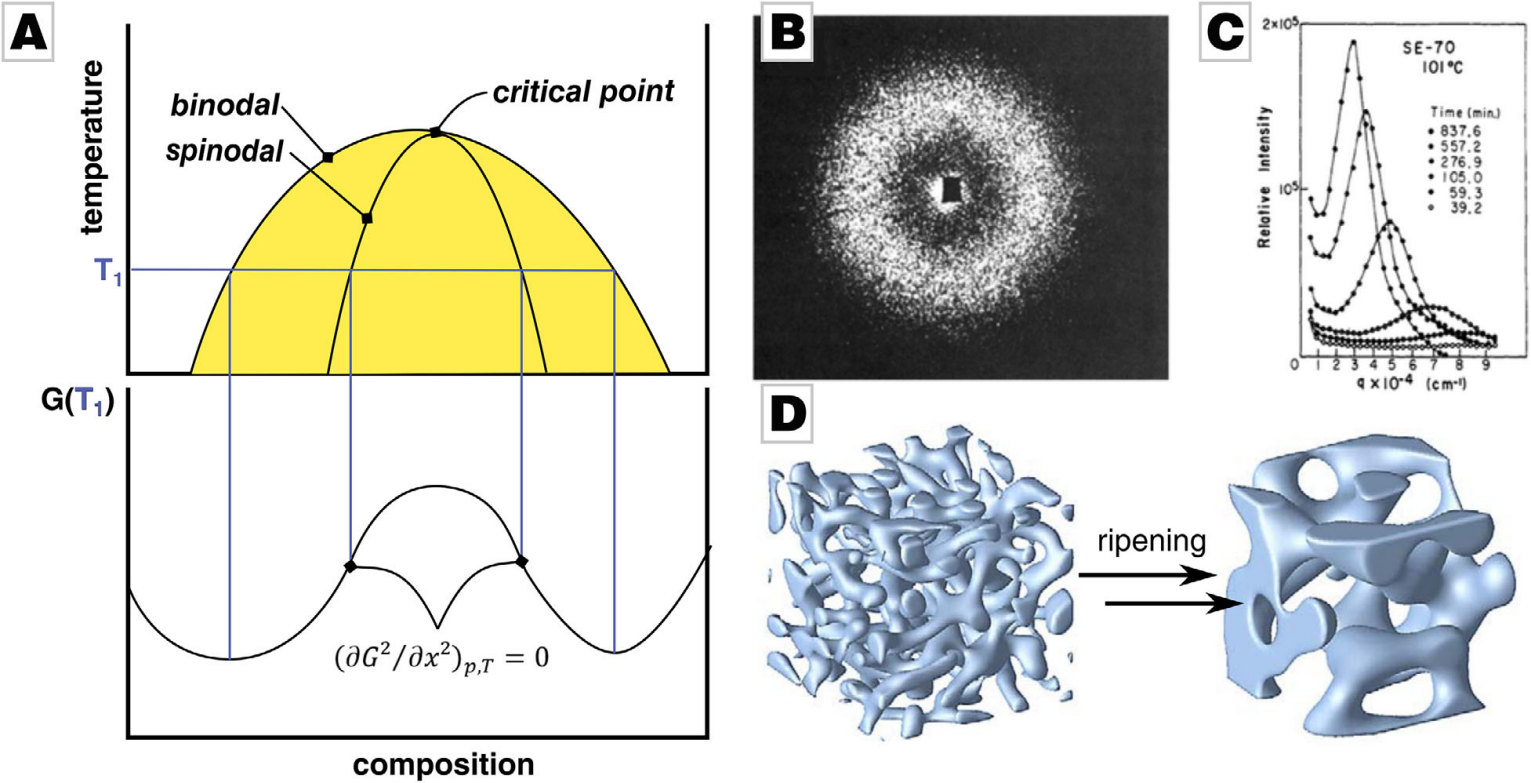
(A) Construction of the spinodal line and the binodal/coexistence line. The locus of the spinodal is given by ${\left(\partial G^{2}/\partial x^{2}\right)}_{p,T}=0$. (B) Light scattering of a demixed polymer blend [58] highlighting the isotropic characteristics. (C) Temporal scattering intensity evolution of a polymer blend, showing only minor changes in the spatial characteristics accompanied by an intensity increase with time [53]. (D) Phase field simulations of the coarsening of a spinodal bicontinuous network with time. Subfigures reproduced with permission from (B, C) Higgins and Cabral [51]. Copyright 2020, American Chemical Society; (D) from *Acta Materialia* 51 (17), Seol et al. [59]. Copyright 2003, with permission from Elsevier.

***Spinodal decomposition*** (SD) is described by Gibbs's second scenario, in which a two‐component system separates because the homogeneous phase becomes not only metastable, but unstable. Then, even small concentration fluctuations may generate new phase domains. For the barrier to vanish, a system has to cross the *spinodal line* located within the binodal region; this line delimits the region of instability against phase separation. Its locus is given by ${\left(\partial G^{2}/\partial x^{2}\right)}_{p,T}=0\;$, and it touches the binodal line at the critical solution temperature (Figure 4A). Upon crossing the spinodal, the homogeneous system immediately undergoes spontaneous clustering, a process unopposed by thermodynamic barriers. The *demixing* starts from infinitesimally small and ever‐present concentration fluctuations whose formation decreases the system's free energy, fueling further uphill diffusion and generating solute‐depleted and solute‐enriched volumes. Initially, these volumes are areas of locally enriched solute, without a phase boundary; thus, no surface energy term opposes demixing. Formation of such solute‐enriched regions inevitably causes the fluctuation waves to propagate throughout the entire volume immediately. Spinodal demixing is therefore always a long‐range process and, in its conception, adheres to the Szilard postulate, since the diffusing and demixing species are presumed to be monomeric (i.e., ions/atoms or individual molecules). Ultimately, two intertwined and reticulated network structures may develop globally throughout the volume. Both networks are bicontinuous, periodic, isotropic, and feature a characteristic length correlating with the fastest spinodal fluctuation wave. If early stages are not arrested (e.g., due to quenching or glass lines), the intertwined networks collapse either into droplets of the minority phase dispersed in the majority phase or the networks morphologically ripen, similar to Ostwald ripening (vide infra) as described by Siggia [43].

Spinodal processes are mostly observed in colloidal soft‐matter systems (e.g., protein solutions, polymer blends) or metal alloy systems, thus, in systems in which the spinodal line can be easily crossed experimentally and in which diffusion is slowed down. For small, low‐molecular‐weight solutes—whether organic (e.g., amino acids) or inorganic (e.g., mineral solutes), especially in aqueous solution—spinodal demixing has not yet been observed in situ. The reason is that burst nucleation intervenes as the system approaches the spinodal line: as supersaturation gradually increases, both the nucleation barrier and the induction time decrease and ultimately vanish at the spinodal line [44]. SD was first observed and interpreted for alloys in 1940 [45] and 1954 [46], respectively. In 1958, Cahn and Hilliard provided the now‐famous evolution equation [47, 48]; the solution of this differential equation mathematically rationalizes the emergence of the emblematic uniform, interpenetrating morphology and the requirement for extended volumes. The bicontinuous stage arises because only sinusoidal composition fluctuations of specific wavelengths λ_m_ proliferate, defining the evolving microstructure [49]. This equation also revealed further hallmarks of SD [48, 50, 51]: Only the concentration amplitudes of specific sinusoidal fluctuations grow, while their spatial wavelength λ_m_ stays constant. Thus, under idealized conditions, spatial features of the evolving microstructure are constant; it is only the local solution composition that changes. Ideally, there is no growth process. The sinusoidal composition fluctuations are isotropic; thus, the phase separation pattern is isotropic but random (Figure 4B). SD occurs throughout the entire sample volume except for near structural/compositional imperfections. Apparently negative diffusion coefficients are found, i.e., uphill diffusion towards regions of high concentration occurs. All of these traits help to trace SD in various systems—be it by negative diffusion coefficients [49, 52], by well‐defined and isotropic scattering behaviors [51, 53] (Figure 4C), or by markedly regular or “worm‐like” morphologies [10, 54, 55, 56] (Figure 4D). Although the latter are perceived as a hallmark of SD, occurrence of small worm‐like morphologies is insufficient to evidence spinodal processes [51] as they can be feigned by insufficient mixing or experimental artifacts [57]. Moreover, as the initially bicontinuous morphology produced by SD usually evolves rapidly into isolated droplets, it becomes indistinguishable from droplet morphologies formed binodally, via nucleation. Thus, SD is particularly difficult to trace experimentally, since the occurrence of a liquid phase or a worm‐like state alone is not sufficient evidence.

**FIGURE 5:**
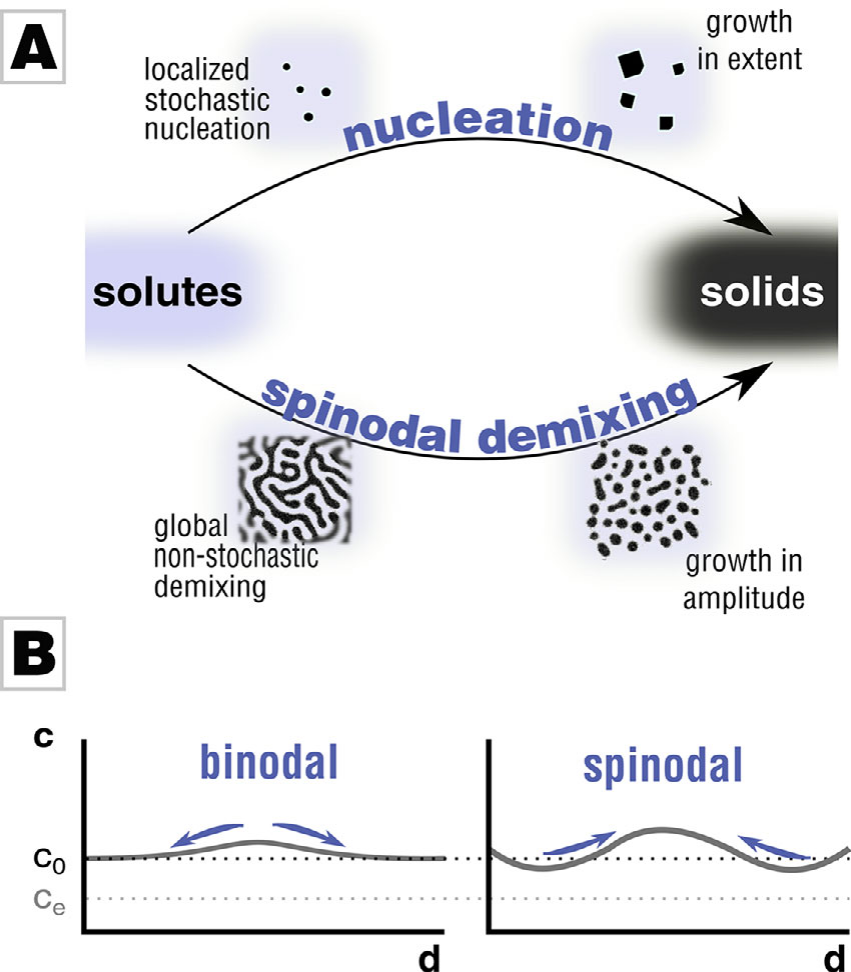
(A) A contrasting juxtaposition of nucleation and spinodal demixing. (B) In nucleation processes, Fick's law is obeyed, and concentration fluctuations in the form of subcritical clusters are attenuated by down‐hill diffusion. In spinodal processes, the phase‐separating species diffuse towards volumes of higher concentration; hence the homogeneous parental phase demixes.

When **contrasting nucleation and spinodal decomposition**, it becomes clear that both processes are diametral opposites in their key characteristics, traits, and preconditions (Figure 5A). Nucleation is a highly localized and stochastic process, characterized by induction times and favored by heterogeneous features of the system (e.g., heterogeneous interfaces, but also in two‐step nucleation). By contrast, spinodal demixing is an immediate, long‐range process without lag time, occurring in a sufficiently extended and homogeneous volume in which concentration fluctuations of sufficient spatial extent can spontaneously grow. Nucleation is strongly controlled by interfacial energy, since CNT describes the formation of a new phase together with its interface. In spinodal demixing, by contrast, interfacial energy is initially negligible, permitting the formation of highly interpenetrating, large‐surface structures; surface‐energy‐driven ripening becomes important only later, once coarsening gives rise to domains sufficiently distinct that phase boundaries—in the sense of abrupt changes in system parameters between phases defined by macroscopic properties—can be meaningfully identified. Downhill diffusion antagonizes nucleation, whereas uphill diffusion drives spinodal demixing (Figure 5B). Nucleation advances by particle growth, while spinodal processes initially progress by localized increase in concentration and not expansion in size. Growth, i.e., coarsening of spinodally‐generated two‐phase systems, occurs subsequently or in parallel to the demixing process (see Figure 4C,D).

### 2.3 Growth and Ripening Processes

*Crystallization* is the formation of a crystalline solid from a parental phase and involves both crystal nucleation and subsequent *crystal growth*. Note that *precipitation* is not limited to the formation of crystalline phases, but also includes the formation of solid amorphous phases [3, 4]. A *crystal* is defined, according to IUCr, by its prominent long‐range ordered arrangement of constituents (ions, atoms, or molecules) *or* by a sufficiently sharp diffraction pattern [60]. A crystal may form through primary, secondary, or heterogeneous nucleation. By contrast, in classical scenarios, spinodal decomposition can generate only liquid or solid amorphous phases, which may subsequently evolve into crystals, for example through two‐step pathways.

Crystal growth from solutions requires two steps: first, transport of the growth units to the crystal surface and, second, their incorporation into the crystal. Both steps, transport and incorporation, may be rate‐limiting and thus controlling process steps in solution‐born processes. The first “classical” concept for crystal growth dates back to the 1920s, when Kossel [61] and Stranski [62] independently provided a kinematic description for the integration processes at solution/crystal interfaces. These models adhere to the Szilard rule that atoms, ions, or molecules incorporate into crystals individually. In their terrace‐ledge‐kink (TLK) model, Kossel and Stranski highlighted the kink (or half‐crystal site) as the energetically preferred site of growth unit integration. Considering the creation of new crystal planes, they introduced the idea that 2d nucleation generates new crystal layers [62]. This model allows for polynuclear growth, where, in the so‐called birth‐and‐spread model, multiple surface nuclei are formed, expand, and merge. Burton, Cabrera, and Frank (BCF) extended the Kossel–Stranski model to spiral growth, driven by screw dislocations that are present in many crystals [63]. The model of a Kossel crystal, although simplified, advanced the understanding of the directional growth rates of crystals.

At low supersaturation, the original Kossel–Stranski model remains valid, but predicts essentially ceasing growth, whereas the more realistic BCF model describes growth on surfaces with active dislocations. The Kossel–Stranski model also becomes inadequate at high supersaturation, where the crystal–solution interface roughens and is no longer atomically flat, contrary to the original Kossel–Stranski picture. In this regime, growth is no longer necessarily governed by incorporation at well‐defined kink sites. Instead, the morphological transformation of the interface indicates a change in growth mode toward so‐called adhesive growth, in which essentially each growth unit reaching the surface is integrated. In a more specific scenario, the roughening transition can be envisioned as steps losing their identity, such that kinks can no longer be attributed to specific steps and the boundaries between steps disappear. The disappearance of terraces may then enforce direct incorporation of solute molecules into kinks, which may lead to slow growth [64]. Mullins and Sekerka provided a morphological instability theory capable of predicting complex interface morphologies, such as cusp and rod arrays or dendritic protrusions, for crystals growing via rough interfaces [65, 66].

*Ripening processes* are outcomes of competing growth processes which are not captured by the above models explicitly. Again, Ostwald broke ground on this matter in two directions. First, he accounted for the greater relative contribution of a particle's surface free energy to its total free energy for small particles as a function of their size *r*, and put forth the Ostwald–Freundlich equation [67, 68] for the increased solubility of small particles *c_s_* (*r*) over that of large crystals, $c_{s}^{\infty }$

(8) $$\ln\frac{c_{s}\left(r\right)}{c_{s}^{\infty }}=2\frac{M\alpha }{\upsilon RT\varrho r}$$

where *M* is the molecular mass, *v* is the molar volume, and ρ is the mass density of the crystal.

**FIGURE 6:**
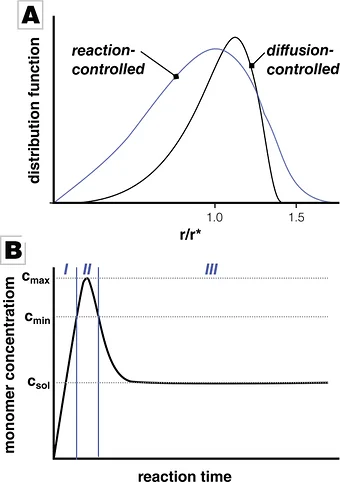
(A) Particle size distribution functions as predicted by Wagner for coarsening under reaction‐ and under diffusion‐controlled conditions. Adapted from ref. [70]. (B) The Lamer model postulates the development of monomer concentration during nanoparticle formation. In stage I, the monomer concentration increases linearly; in the original work of LaMer and Dinegar, soluble monomers form by a chemical reaction. Stage II commences when nucleation starts, owing to reaching the hypothetical threshold supersaturation for nucleation *c*_min_. In stage III, nucleation ceases, and particle growth reduces the monomer concentration to its solubility limit *c*_sol_. See refs. [72, 77] for more details. Subfigure (B) adapted with permission from Whitehead et al. [72]. Copyright 2019 American Chemical Society.

In Ostwald ripening, larger particles grow, feeding on smaller particles, which dissolve owing to the excess of surface free energy [68]. In 1961, the kinetics of this mode of particle coarsening was treated quantitatively by Lifshitz and Slyozov [69], and Wagner [70] (Figure 6A).

The LaMer model (Figure 6B) was introduced by LaMer and Dinegar in 1950 to explain the formation of monodisperse particle sols from continuously generated solute [71, 72]. It attributes the narrow particle size distribution to *burst nucleation*, that is, a very high nucleation rate over a short period of time. Because the particles form nearly simultaneously, they initially exhibit similar sizes and thus similar curvatures, which reduces the driving force for subsequent Ostwald ripening and thereby helps maintain a comparatively narrow size distribution.

**FIGURE 7:**
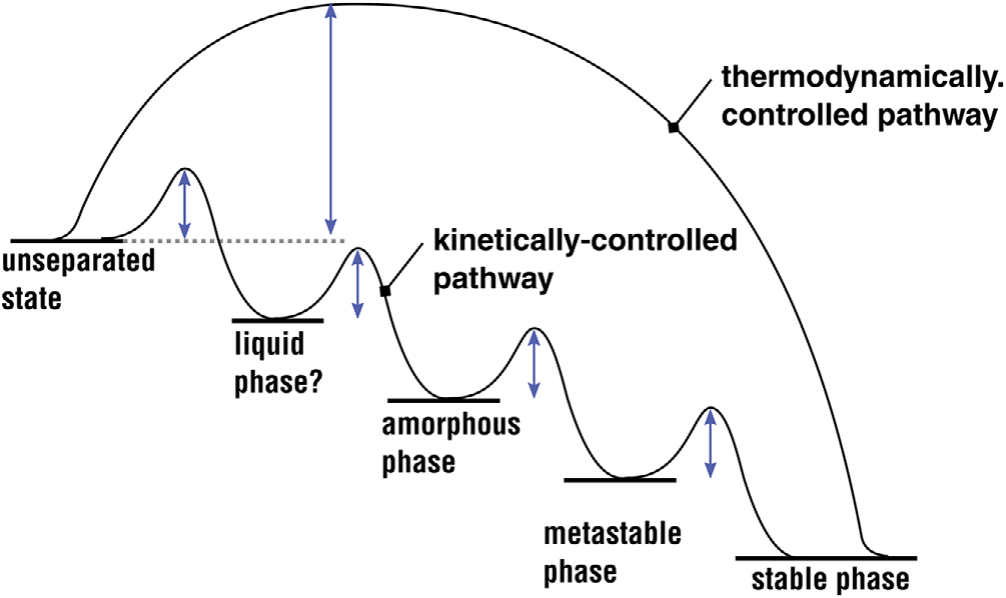
Ostwald's step rule predicts that in a kinetically controlled series of transformations, the next phase to form is also the nearest one. Under kinetic control, a reaction pathway is selected with minimal activation barriers (in blue), in contrast to the direct transformation pathway. Ostwald even expected the separation of a liquid phase before a solid phase is formed, due to the low interfacial energy of a liquid/liquid phase boundary. Foreign components can drastically alter the selected pathway [74]. Adapted from Cölfen and Mann [78], Copyright 2003, with permission from Wiley‐VCH.

Ostwald also considered the competition between phases in a polymorphic system and their chronological appearance. He postulated that “when leaving any state and transitioning to a more stable one, not the most stable state under the existing conditions is sought, but the closest one”^1^ [73], see Figure 7. He stated clearly that this now‐called *Ostwald's rule of stages* (or *Ostwald's step rule*) is not strict and violations are possible, especially in a multicomponent system (e.g., when foreign ions are present [74]). The rule can be rationalized by considering phase‐specific nucleation rates: the height of the nucleation barrier $\Delta G_{N}^{*}$ strongly depends on the free energy term and, specifically, on the interfacial tension ($\Delta G_{N}^{*}\propto \alpha^{3}$, see Equation 5 and Figure 3). Since less stable polymorphs often feature lower interfacial energy [75, 76], they require lower critical radii (r*∝α) and, most important, achieve much higher nucleation rates (see Equation 4). Ostwald's step rule was derived later also via irreversible or statistical thermodynamics.

## 3 Nonclassical Developments

### 3.1 Nonclassical Nucleation Processes

#### 3.1.1 Nonclassical Nucleation Processes: Two‐Step and Multi‐Step Mechanisms

CNT assumes that nuclei form by sequential association of single molecules/ions from a supersaturated phase. This assumption is equivalent to assuming that the parental phase is homogeneous without any internal structure and the concentration fluctuations that lead to new phase nucleation are only driven by thermal motions; concentration fluctuations are enhanced if the solute molecules exert short‐ or long‐range attraction [79]. In 1997, ten Wolde and Frenkel predicted the first partial deviation from the classical scenario [80]. They carried out numerical simulations of solutions, for which a liquid–liquid critical point is submerged below the liquidus line in the phase diagram, i.e., liquid–liquid separation occurs in solutions supersaturated with respect to crystals. Examples of such systems include protein solutions, some colloid suspensions, and selected solutions of large organic molecules. The concentration fluctuations around the liquid–liquid critical point are enhanced [81, 82]. Ten Wolde and Frenkel predicted that in the region of enhanced critical concentration fluctuations the barrier for crystal nucleation is diminished [80], a conclusion later supported by computational and analytical works [83, 84].

The great value of the ten Wolde and Frenkel analysis was that it suggested for the first time that the concentration and structure fluctuations necessary to create an ordered crystal nucleus do not have to occur synchronously, as implicitly assumed in CNT, but the concentration fluctuations can precede structure formation. Notably, crystal nuclei still form by sequential association of monomers in a region of enhanced monomer concentration, as in the classical Volmer mechanism. There are several limitations of the ten Wolde and Frenkel mechanism. The particular phase diagram that ten Wolde and Frenkel assumed is rare for small‐molecule organic and inorganic systems. Furthermore, for systems with the required phase diagram, the critical point for liquid–liquid separations is often submerged below the gelation line, i.e., the solution gels and fully stalls crystal nucleation [85]. Lastly, even though the critical fluctuations are longer‐lived than thermal fluctuations, their lifetime may still be too short for crystal nuclei to form. Not surprisingly, there appears to be no experimental confirmation of crystal nucleation supported by critical concentration fluctuations.

Haas and Drenth proposed that crystals nucleate not in the critical region for liquid–liquid separation, but below the line of coexistence of the two liquids, within the dense liquid droplets [86]. Within the droplets of the dense liquid phase, the solute was suggested to be at a higher supersaturation than in the solute‐depleted parental phase, which would significantly increase the nucleation rates. The latter assumption is unphysical: dense liquids equilibrate with the host solutions fast and, in equilibrium, the solute chemical potential in the dense liquid is equal to that in the dilute solution. Still, nucleation hosted by dense liquids is possible if the dense liquids do not gel and retain low viscosity, as with deoxy sickle cell hemoglobin [87, 88, 89, 90, 91]. The driving force for the preferred nucleation within the dense liquids is likely the lower surface free energy of the crystal nucleus in contact with the dense phase (cf. Figure 3) [29].

Note that crystal nucleation hosted by dense liquids diverges from the scenario assumed in the Ostwald step rule by the active role that the dense liquid plays in the nucleation of the more stable phase, the crystal. Importantly, the high concentration of the dense liquids often leads to gelation, with complete cessation of nucleation in the resulting gels. Thus, crystallization practitioners follow the rule that as soon as liquid–liquid separation is observed, which they often refer to as *“oiling out*”, crystallization would be impossible. This is consistent with observations of binodal liquid‐condensed phase formation in mineral solutions, for example MCO_3_ (M = Ca, Sr, Ba, Mn, Cd, Pb) [8, 10, 92] and cerium oxalate [56, 93].

**FIGURE 8:**
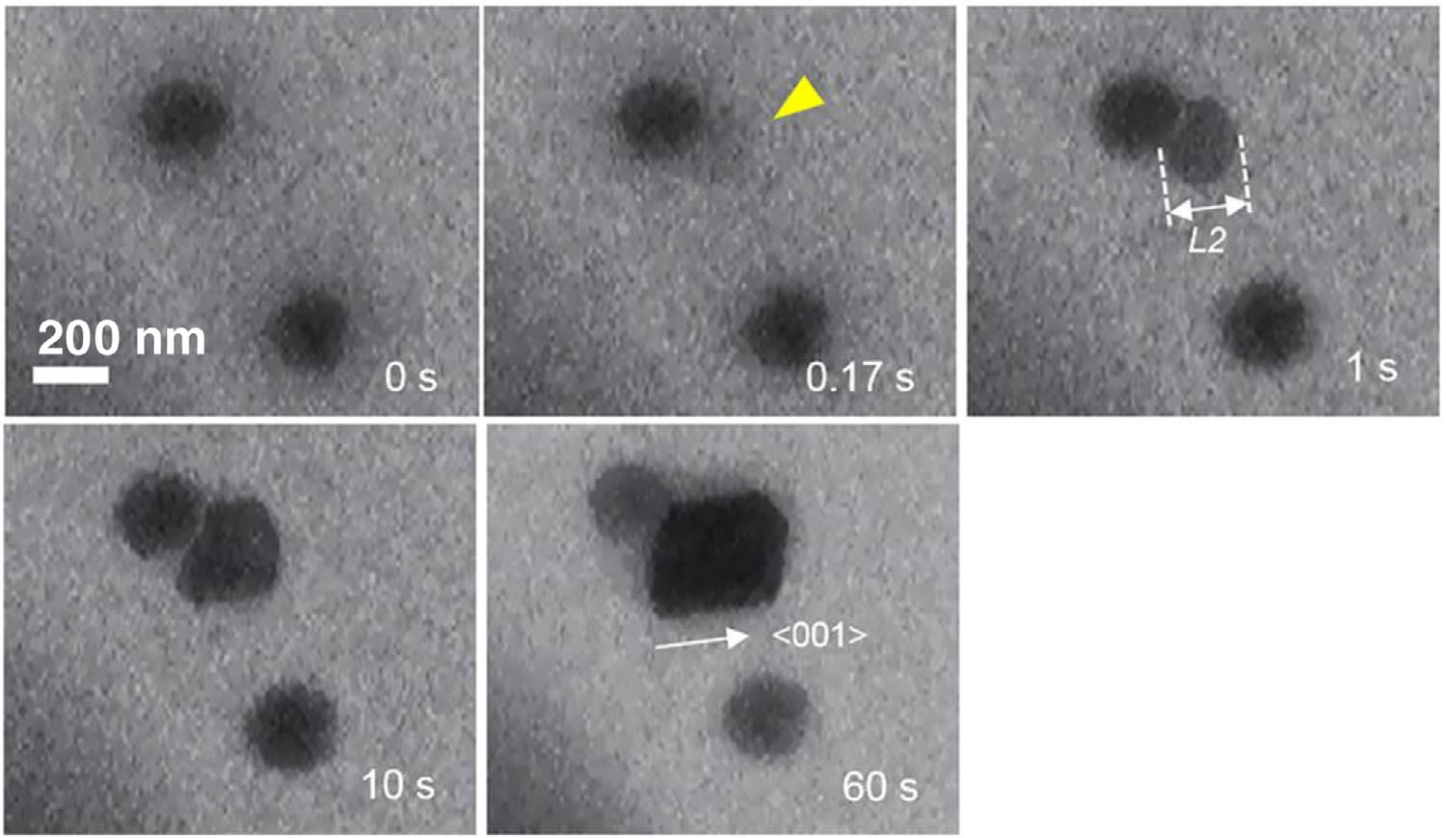
Time‐resolved in situ TEM of lysozyme crystal nucleation shows formation of a spherical particle (yellow arrow) in the vicinity of an amorphous solid nanoparticle that already solidified. The new droplet transforms into an orthorhombic crystal. Reproduced from Yamazaki et al. [98]. Copyright 2017, National Academy of Sciences.

The first experimental evidence of nonclassical crystal nucleation came from careful measurements of the homogeneous (i.e., unaffected by foreign particles or molecules) nucleation rates of crystals of the protein lysozyme [28, 85, 94]. These tests revealed correlations of the nucleation rate with the supersaturation, *J*(Δμ), which were not smooth, and temperature, *J*(*T*), which were nonmonotonic, both in striking contrast to the CNT predictions. The broken *J*(Δμ) dependences indicated discrete transitions between very small nucleus sizes, from 10 to 4 to 1 molecule. In turn, the small nucleus sizes implied, according to Equation (5), that the surface free energy experienced by the nuclei is low, about 10‐fold lower than that measured for the interface between a crystal and the solution. The nonmonotonic *J*(*T*) consisted of an intuitive increasing section as *T* is lowered away from the solubility line, increasing Δμ, and a surprising decreasing section at further lowering of *T*. The decreasing branch suggested a strongly temperature‐dependent and high viscosity of the medium, in which the crystal nuclei form. Importantly, the probed phase regions were away from the region of liquid–liquid coexistence. Taken together, these three pieces of experimental evidence indicated that crystal nucleation occurs within precursors that share certain properties with the dense liquid but are distinct from it [95, 96, 97]. Remarkably, the mechanism deduced from the nonclassical *J*(Δμ) and *J*(*T*) dependences was validated by direct observation of lysozyme crystal nucleation within dense liquid clusters by liquid phase and cryo‐EM [98], see Figure 8.

Liquid–liquid separation also plays a role in phase change processes, as shown for structural rearrangement of colloidal 2d crystals that transition via a liquid intermediate [99]. Even if the dense liquids are metastable with respect to crystals, they do not necessarily induce crystal nucleation. They also may undergo solvent expulsion processes, i.e., dehydration, yielding *solid‐amorphous phases*, as observed in protein [98] and mineral solutions [8, 92, 100]. Such solid amorphous phases can also form via direct nucleation, without a prior liquid phase. Solid amorphous phases are metastable against a crystalline phase and, thus, may also host the formation of a crystalline phase within the amorphous matrix, a scenario akin to two‐step nucleation. One of the first reports stems from van Mergen and co‐workers, who studied a colloidal model system featuring an amorphous phase [101] hosting crystal nucleation [102]. Note that diffusion in solids is significantly lower than in liquids, and thus two‐step nucleation in a solid precursor phase may act on different time scales.

**FIGURE 9:**
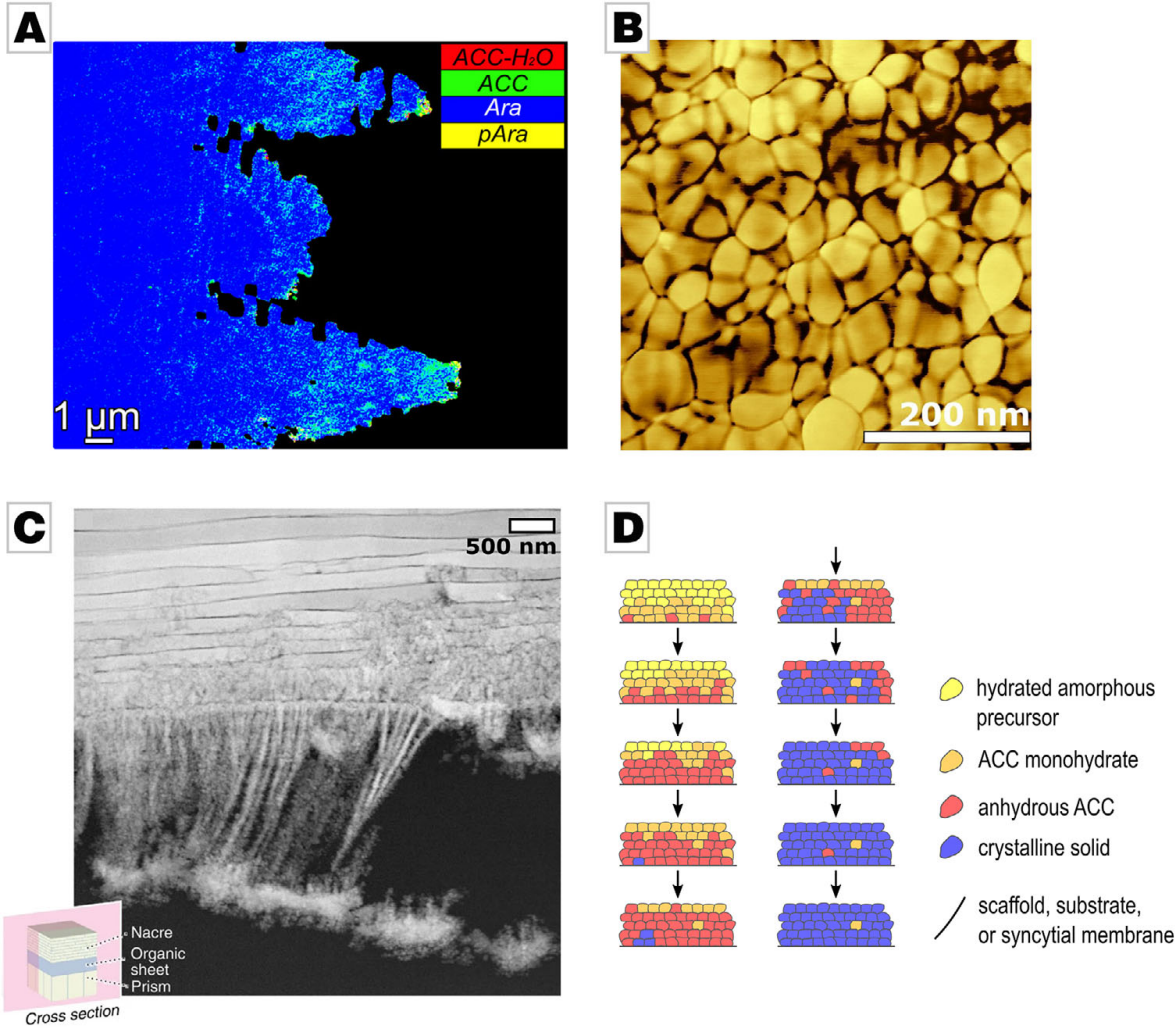
(A) XANES‐PEEM mapping of nacre (red abalone, *Haliotis rufescens*) [109], with the color code given in the inset. The amorphous precursor is proto‐calcitic ACC, although the mature nacre is composed of aragonite. Islands of amorphous materials are still present well beyond the growth front. (B) Atomic force micrograph (tapping mode) of an archetypal nanogranular biomineral [134], here calcite prisms of *Pinna nobilis* [111]. (C) Annular dark‐field scanning electron micrographs of the onset of nacre in *Pinna nobilis* [149]; nacre forms by nanoscale particle aggregation with particle sizes coherent with that of the nanogranular fine structure [134]. (D) Schematic representation of how crystallinity may hypothetically percolate through an amorphous precursor during transformation; one possible transition scenario in calcium carbonate is illustrated [134]; see also Weiner and Addadi [150]. Other putative pathways have likewise been proposed; see Gower (2025) [132] and references therein. Reprinted from (A) DeVol et al. [109]. Copyright 2015, with permission from American Chemical Society, (B) Wolf et al. [111]. (C) Hovden, Wolf and co‐workers [149], (D) Wolf et al. [134]. Copyright 2016 with permission from Elsevier.

Two‐step processes involving solid‐amorphous phases have been reported for various systems [103, 104], most prominently in biomineralization (see Glossary). The occurrence of biogenic ACC was reported already in, e.g., the 1950s by Odum for nudibranch spicules [105]; the works of Lowenstam and co‐workers then showed the broader occurrence of amorphous phases in biomineralization [106]. A ground‐breaking series of works by Addadi, Weiner, and co‐workers revealed that transient amorphous calcium carbonate (ACC) serves as a harboring precursor for calcite formation in sea urchin larval spicules [107, 108], shaping the morphology and properties of the mature biomineral. Similar pathways were later recognized for a large number of mineralizing tissues [109, 110, 111], including enamel [112], fish bone [113], corals [114], or bivalve shells [111, 115, 116]. After nucleation of a crystalline phase in the harboring amorphous solid, e.g., by heterogeneous nucleation by templating interfaces [117], the crystallization front percolates through the solid body, a process usually studied by synchrotron‐based techniques [109, 110, 115, 118] (e.g., XANES‐PEEM, Figure 9A). In these systems, extensive re‐dissolution of the amorphous body is largely suppressed by various, partly unresolved mechanisms, so that the final stage of crystallization proceeds via a shape‐preserving phase transformation: crystallinity propagates through the amorphous precursor body while retaining its morphological features (Figure 9D). In geomineralogical terminology, it is therefore correct to describe such shape‐preserving transformations as *pseudomorphic* when the chemical composition changes, whereas *paramorphic* is reserved for cases in which the composition remains unchanged.

**FIGURE 10:**
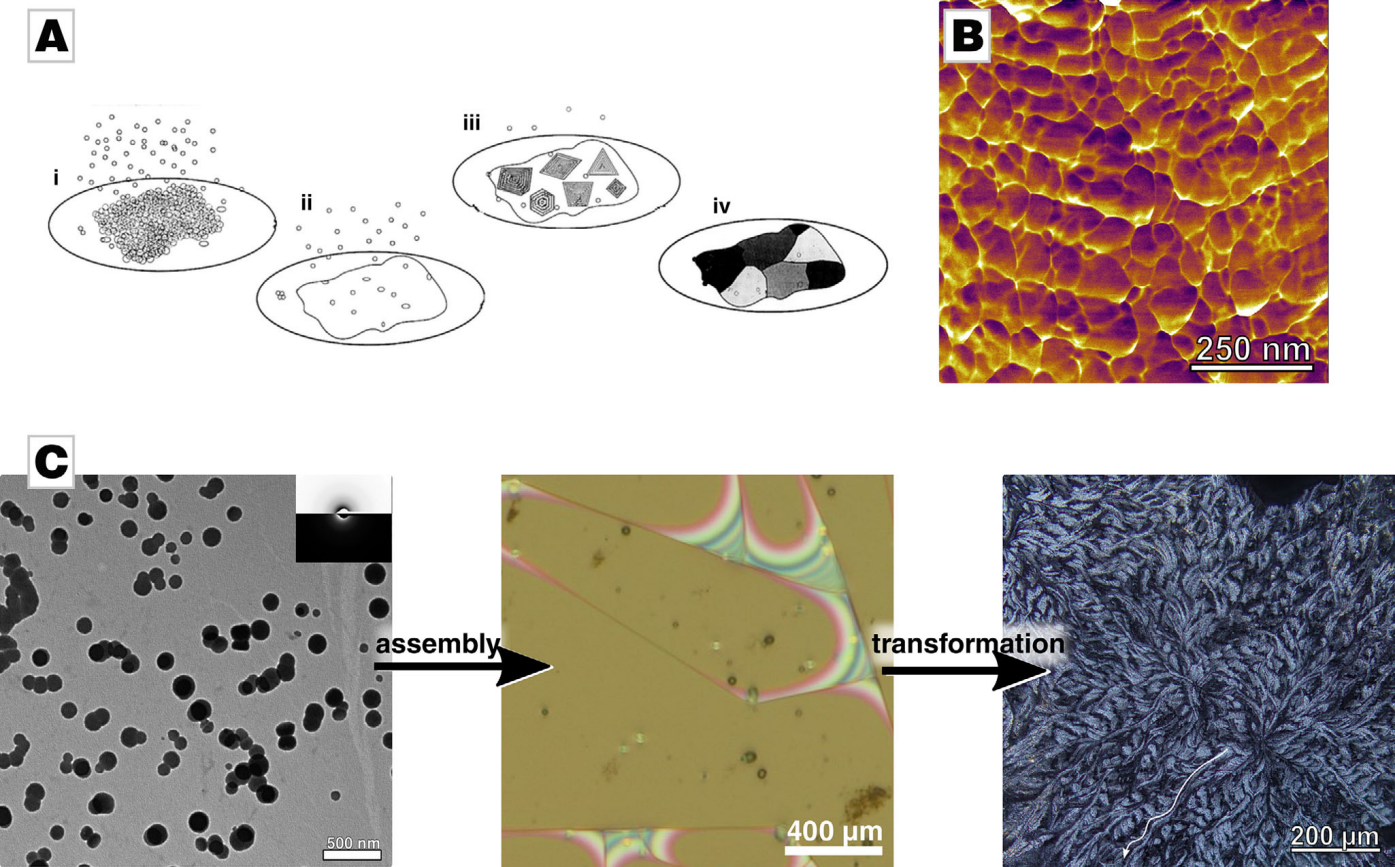
The polymer‐induced liquid‐precursor (PILP) process: (A) first conceptual scheme by Gower, (B) nanogranular ultrastructure traced by phase‐modulated AFM, compare with Figure 9B. (C) Experimental examples of the individual stages of a PILP / CAT process. Colloids form and assemble into an amorphous body, here a thin film, that subsequently transforms into a poly‐/mesocrystalline state under preservation of the macroscopic shape. Left: standard TEM of highly hydrated ACC droplets stabilized by the protein ovalbumins. Middle: amorphous thin film generated with polyacrylic acid, images by polarized light microscopy which highlights stressed regions caused by dehydration. Right: spherulitic growth patterns in a transformed PILP film, featuring so‐called crystal lattice tilting. Reprinted from (A) Gower and Odom [126], Copyright 2000, with permission from Elsevier; (B, C right) Harris et al. [151]. Copyright 2015, published by RSC and reproduced under terms of the CC‐BY license; (C left) Wolf et al. [135]. Copyright 2011, with permission from American Chemical Society.

A fundamental question in biomineralization research is to identify key regulatory factors that control mineral formation, transient precursor cascades, and the emergence of mineralized tissue architectures [119, 120, 121, 122, 123, 124]. Mineralization‐regulating proteins in calcifying organisms typically exhibit unusually low isoelectric points, arising from carboxyl‐rich primary sequences, low‐complexity repeat motifs, and/or acidic post‐translational modifications [125]; their activity was often rationalized within prevailing classical frameworks, primarily as crystal‐growth inhibition or substrate templating [125]. The pivotal synthetic impulse came from Gower's early graduate work, first presented as a poster talk at the 1995 Gordon Research Conference on Biomineralization and later published with Odom [126]. She demonstrated that polyanionic additives can induce polymer‐stabilized amorphous calcium carbonate precursor phases and non‐equilibrium calcite morphologies (Figure 10), including mineral films emulating thin nacre tablets [126, 127, 128]. Subsequent work by Gower and co‐workers on the long‐sought intrafibrillar mineralization of collagen further highlighted the relevance of additive‐stabilized precursor pathways [129, 130, 131]. These findings helped spur the broader search for transient amorphous precursors in biominerals and for key regulatory factors capable of stabilizing or processing them [132].

Due to the liquid‐like intermediate stages, Gower and co‐workers suggested the formation of a transient liquid‐condensed mineral precursor phase [126, 133], the *polymer‐induced liquid precursor* (PILP), see Figure 10A. PILP‐mediated mineralization processes are thought to progress via multiple stages. After separation of the PILP precursor (for which it is not yet established whether it is a true phase or more of a solute aggregate or associate), the precursor droplets assemble or coalesce into a larger mineral precursor body, which first solidifies by dehydration and, then, may transform pseudomorphically into a crystalline material with pronounced non‐equilibrium morphologies (Figure 10C). To this day, this polymer‐controlled mineralization process remains unique in reproducing the nanogranular ultrastructure of biominerals (compare Figure 10B with Figure 9B), one of the characteristic hallmarks of biogenic minerals from which numerous structure–property relationships emerge (see ref. [134] for more details). The process is, thus, a two‐step nucleation process, but although a liquid intermediate occurs, nucleation takes place in the solidified amorphous phase. These droplets have been visualized by standard [111, 135] (Figure 10C, left) and cryogenic [136, 137] TEM studies, and in situ AFM determined a modulus consistent with a densifying liquid‐like phase [138]. The PILP mechanisms seem not to be restricted to biomineral‐associated minerals (such as calcium carbonate and phosphate) since similar multi‐step processes have also been reported for other systems [139, 140].

The term *colloid assembly and transformation* (CAT; see Glossary), introduced by Wolf and Gower [141], broadens the PILP concept beyond its restriction to a truly liquid precursor phase. Instead, CAT emphasizes the assembly of nanoscale colloidal amorphous precursor entities into a larger precursor body and its subsequent transformation, while allowing the precursor body to pass through liquid‐like, viscoelastic, gel‐like, partially coalesced, and progressively densifying states. Later work by Gower and Elias particularly emphasized the viscoelastic character of PILP‐type precursors, consistent with this broader terminology [142]. This distinction between CAT and PILP also separates implicitly the existence of a non‐Szilard/CAT‐type precursor pathway from the preservation of an overtly nonclassical final morphology. Polymers are not a necessary condition for such pathways, but they are particularly efficient process‐directing agents because they can simultaneously stabilize, accumulate, assemble, and compartmentalize precursor entities, while suppressing redissolution, ripening, or maturation toward fully crystalline products. Small molecules, ions, solvent components, confinement, or suitable kinetic process control may achieve related effects, although often in a less combined or less robust manner. Thus, additive‐/polymer‐free systems may pass through or populate non‐Szilard precursor states and still yield classical‐looking crystals, whereas additive‐ or process‐stabilized systems more readily preserve molded, pseudomorphic, nanogranular, or otherwise non‐equilibrium morphologies.

With the identification of proto‐structured amorphous phases, a special twist came into two‐ and multi‐step processes. Especially in the case of ACC, variations in the near‐range order of ACC were found both in vitro and in vivo [143, 144, 145, 146], with structural similarities to crystalline calcium carbonate polymorphs [144]. This so‐called polyamorphism [147] triggered the idea of *protocrystalline structures*, i.e., proto‐ in the sense that the polyamorphous near‐range order may predetermine the next crystalline phase to form. However, various reports showed that this is not necessarily the case [148]; for example, XANES‐PEEM mapping of nacre (Figure 9A) points to proto‐calcitic ACC as the amorphous precursor of crystalline aragonite [109].

#### 3.1.2 Nonclassical Nucleation Processes: Prenucleation Clusters

A hypothetical equilibrium distribution of cluster populations along the nucleation free‐energy landscape would follow the Boltzmann law, resulting in very few critical clusters. CNT holds that beyond the critical size, larger new‐phase domains have lower free energy and therefore continue to grow. Extending the same equilibrium description beyond the barrier would therefore require the reverse process, i.e., the dissolution of growing particles into smaller ones and, ultimately, into critical clusters — a process that is impossible in a supersaturated solution [19].

The nucleation precursors, hypothesized in nonclassical nucleation mechanisms, were expected to occupy a metastable minimum in the evolution of the system's free energy along a reaction coordinate that first moves from low to high density and then from disorder to crystalline order [89, 95, 152, 153, 154]. In protein solutions (e.g., hemoglobin or lumazine synthase), light scattering experiments identified these precursors as a new phase with unique properties: the domains of this phase were about 100 nm in diameter, their size distribution was narrow and centered at the average size, this size was steady in time, and, most unusually, independent of the solution concentration [89, 155, 156, 157]. The precursors were initially referred to as metastable dense liquid clusters—their fluidity was certified by time‐resolved in situ AFM—but to avoid misidentification as droplets of a solute‐rich liquid, they were later called *mesoscopic solute‐rich clusters*. Importantly, the clusters exist with identical properties in solutions that are both supersaturated and undersaturated with respect to crystals. Clusters with the same signature characteristics have by now been observed in solutions of numerous proteins [87, 89, 155] and organic molecules [158, 159, 160]. The surprising features of the clusters were directly visualized by liquid‐phase and cryo‐electron microscopy and atomic force microscopy [89, 157, 159, 161, 162, 163]. Theoretical efforts to understand the molecular mechanism of formation of clusters with such unusual behaviors propose that they exist owing to the accumulation of transient solute dimers [164, 165, 166, 167, 168]. The cluster size is determined by the balance between the lifetime of the transient dimers and their rate of outward diffusion from the cluster core. The amount of solute captured in the clusters, and the related number of clusters and cluster population volume are governed by thermodynamic equilibrium between the clusters and the bulk solution, which enforces a quasi‐exponential increase of the number of clusters with the solute concentration [167].

**FIGURE 11:**
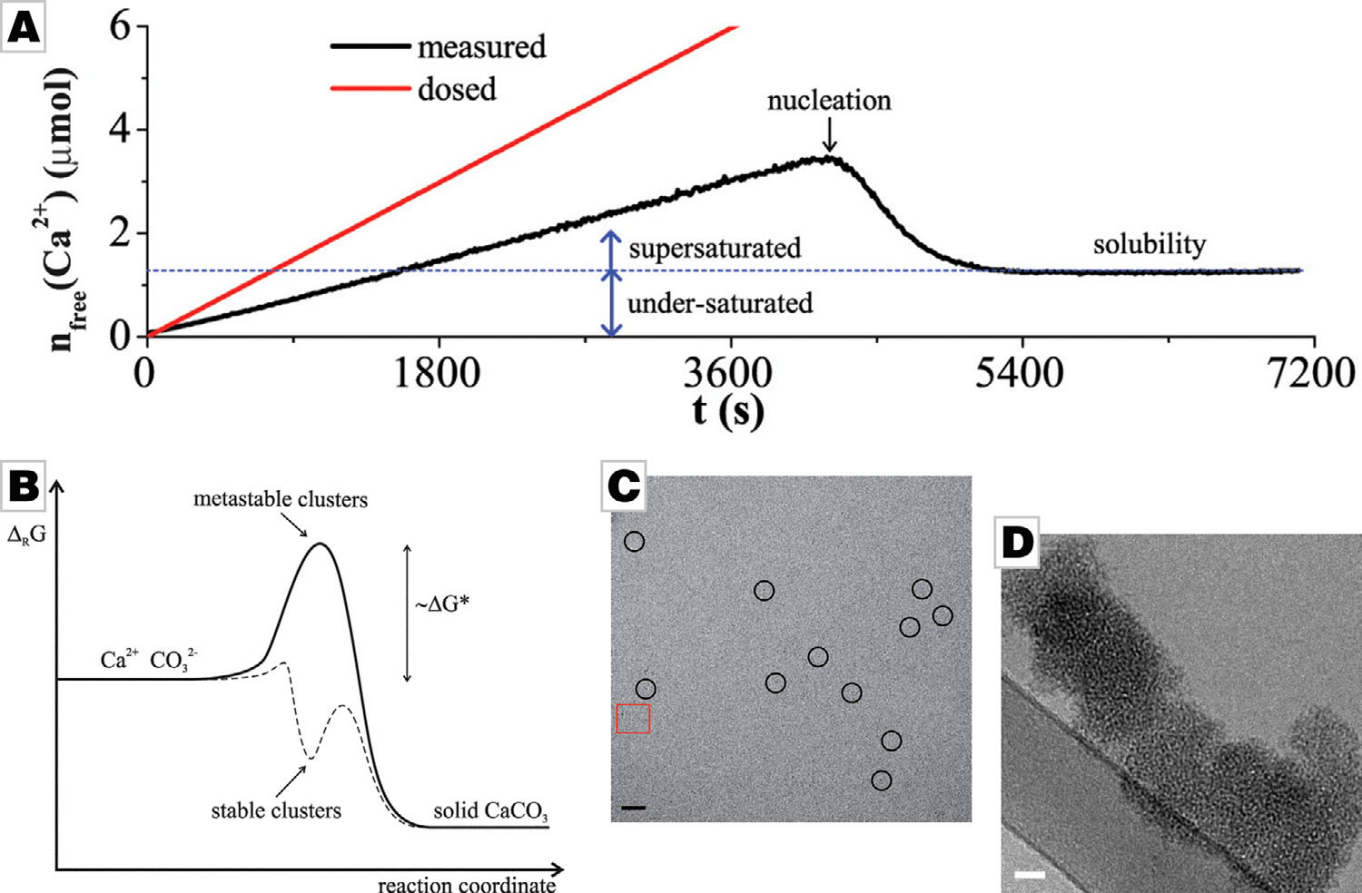
Prenucleation cluster formation in the calcium carbonate system (A) Calcium activity evolution with increasing Ca^2+^ concentration accomplished by steady dosing at constant pH; the increase is thus represented by the time axis. The red line denotes the dosed ions, while the black line represents the Ca^2+^ concentration evolution measured by a calcium‐selective electrode. (B) Proposed reaction free energy Δ_R_G variation during calcium carbonate formation, assuming PNC involvement. Classically, the nucleation barrier must be overcome (bold). By contrast, PNCs are trapped in a metastable local minimum; their formation barrier is thought to be negligible compared to thermal energy. (C,D) CryoTEM micrographs initially interpreted as showing nanoparticulate precursors during heterogeneously controlled calcium carbonate formation; left in absence of additives, right in presence of poly(aspartic acid). Reproduced from (A, B) Gebauer et al., Science, 10.1126/science.1164271 (2008), (C) Pouget et al., Science, 10.1126/science.1169434 (2008), AAAS, (D) Xu et al., Nat Commun. 10.1038/s41467‐018‐05006‐w.

Inspired by the mesoscopic protein‐rich clusters, in 2008 Gebauer et al. reported so‐called stable prenucleation clusters (PNCs, see Glossary) in aqueous solutions of calcium carbonate, a mineral‐forming inorganic substance. These nanoscale supra‐ionic entities represent an ergodically [169] persistent solute population which is stable in size over hours [170]. From ion association equilibria based on operando potentiometry (Figure 11A), a stabilization of about 7 *k_B_T* was determined (Figure 11B). Later isothermal calorimetry and operando potentiometry showed an entropic stabilization [171]; thus, an energy contribution beyond the classical binary energy balance. MD simulations of highly supersaturated solutions by Gale and co‐workers [172] described these clusters as highly dynamic inorganic polymers in which bidentate carbonate ligands interconnect calcium ions. These associates show a constantly‐reorganizing topology of chains, branches, and even rings. Later, they stressed that at low (non‐saturated) millimolar concentrations, the fraction of these species will remain small, at least until the system reaches the binodal [173]. Wolf et al. conducted mass spectroscopy studies of supersaturated calcium carbonate solutions that corroborated these simulations as they showed near‐to equimolar cluster composition [92], in agreement with the compositional features found in MD simulations. In the initial reports on prenucleation clusters, no direct evidence was provided on how and whether these prenucleation clusters are actually involved in later phase separation processes. Pouget et al. provided cryoTEM studies that were interpreted as showing nanoparticle assembly during interface‐controlled, and thus heterogeneous, calcium carbonate formation under supersaturated conditions [174]. Sommerdijk and co‐workers later extended this line of work to polymer‐controlled conditions, directly visualizing the assembly of nanoparticles into larger precursor bodies [136]. (Note that this polymer‐containing case should be distinguished from additive‐free PNC pathways, where comparable precursor states may be shorter‐lived and need not be preserved in the final crystal morphology.) Further arguments invoked the synthesis‐dependent polyamorphism of ACC and its correlation with PNC association equilibria [147, 170, 172]. The latter also leads to the notion that PNCs also feature proto‐structures [147]; however, no experimental evidence is currently available for this claim. The observed clusters challenged CNT, and the lack of unanimously accepted evidence of how these clusters participate in phase separation spurred criticism. Neoclassical interpretations were put forward to circumnavigate the novel PNC concept or the PNC pathway, such as polymerizing ion pairs [175], nanoscopic spinodal‐like demixing [11], or binodal liquid–liquid phase separation without involvement of clusters [176], or generally questioning ion clustering in solution [177]. Indeed, the question of whether supra‐ionic clusters exist also in undersaturated conditions may require further tests, while the formation of supra‐ionic species at elevated concentrations is coherent with classical and nonclassical notions.

Terahertz spectroscopy of calcium carbonate solutions showed a coincidence between phase separation and reduced water dynamics [175], a finding from which the *nonclassical nucleation* mechanism of the prenucleation cluster pathway was derived. In this speculative model, a critical loss in molecular dynamics of PNCs — induced by changes in solution‐intrinsic conditions, although the underlying trigger remains unspecified and possible candidates such as supersaturation, ionic strength, ion activities, or cluster concentration remain putative — causes structural alterations and transformation of PNCs into phase‐separated nanodroplets [175, 178]. These droplets are supposed to coalesce subsequently into mesoscopic droplets, which then crystallize or solidify [179]. This model of nonclassical nucleation opposes CNT in multiple aspects, i.e., (a) this nucleation mode is not induced by the exceeding of a critical size, (b) nonclassical nucleation in the framework of the so‐called prenucleation cluster pathway is then an inter‐correlated, non‐stochastic, global event as it occurs for the entire PNC population upon applying suitable triggers. Currently, further validation of this nonclassical nucleation scheme, identification and experimental verification of specific nonclassical nucleation triggers, and its applicability in other inorganic systems still awaits further progress.

A recent cross‐scale structural analysis of ACC revealed a hierarchical multi‐level organization consistent with a multi‐step aggregation process, i.e., association of building units without full merging, rather than with the previously suggested mesoscopic PNC coalescence, which would imply fusion into a continuous phase with loss of the original interfaces [169, 179]. Employing Monte Carlo simulations constrained by X‐ray and neutron scattering data, Clark, Wolf and co‐workers found 2‐nm‐sized domains, separated by water, as the fundamental building unit of ACC [180], a size in good agreement with observations with PNCs under varying conditions [181, 182]. This structural evidence for phase separation via multi‐scale assembly of discrete nanometric supra‐ionic units, putatively PNCs, is well in line with the early cryoTEM studies of Pouget et al. on interface‐controlled calcium carbonate formation [174]. In subsequent work, Wolf and co‐workers established a labeling strategy for the supra‐ionic cluster species, which also provided chemical evidence (i.e., based on composition) for their involvement in ACC formation [183]. It was found that only those additives that specifically interacted with clusters (by integrating into and even initiating clusters) were later also found in the amorphous phase. Additives not interacting with clusters in solution could not be traced in the final ACC precipitate [183]. The molecularly specific interaction of additives with clusters was later extended to chemically related cation‐exchange mechanisms for foreign cations [184]. Together, these studies provide chemical and ultrastructural support for the notion of ACC being composed of aggregating clusters. Moreover, the study on antiscaling agents was probably the first to reveal a molecular mechanism of nonclassical inhibition of classical nucleation in an inorganic/mineral system. Classically, nucleation inhibition is only feasible by reducing supersaturation, e.g., through chelating agents that reduce ion activity. Here, cluster‐interacting or ‐integrating agents considerably stabilized the solution against phase separation, a behavior akin to nonclassical inhibition phenomena in protein systems [97, 153].

Calcium sulfate forms via a similar aggregation‐based trajectory involving supra‐ionic species, as experimentally shown by Stawsky, van Driesche, and others [185, 186, 187]. Polynuclear coordination clusters (P1) form, in agreement with computational predictions [188]. These evolve into an amorphous “pseudo‐phase” (P2) of nanometric, anisotropic entities of lower dynamics [186, 189]. Upon critical particle number density, P2 units assemble into disordered aggregates (P3), which harbor crystal lattice formation. For this, P2 entities reorganize into first one‐dimensional, then multilayered assemblies, from which micron‐sized gypsum ultimately grows.

For amorphous calcium phosphate (ACP), again cluster aggregation is put forth, but cluster composition also remains unclear. Already in 1974, Betts and Posner inferred from X‐ray PDF that ACP consists of Ca_9_(PO_4_)_6_ nanounits with interstitial water [190] (note the structural similarity to ACC as reported by Clark et al. [180], vide supra), later supported by cryoTEM studies by Dey et al. [191]. However, Habraken and others [192, 193] suggested condensing calcium triphosphate units [Ca(HPO_4_)_3_]^4−^. Recent NMR studies not only supported phosphato‐calcium coordination entities assembling into prenucleation aggregates [194]; they stressed that the atomic structure and composition of these species are sensitive to external factors, such as pH [195, 196].

### 3.2 Nonclassical Mechanisms of Crystal Growth

The classical mechanisms of crystal growth rely on the Szilard postulate, i.e., that molecules associate with the crystal individually and sequentially. Accordingly, any kinetic pathways in which assemblies of solutes form in the solution and incorporate into the crystal as a whole are considered nonclassical. The solute assembly was thought to range from liquid or amorphous particles, or crystallites [197].

The nonclassical mechanism of crystal growth discovered first was *oriented attachment* (OA), a growth mode which is common at (initially) extreme supersaturations in which a crystal grows by the association of preformed nanocrystallites. Like aeolotropic (i.e., directional) interactions of globular proteins [198], oriented attachment features directionally controlled aggregation, which can lead to a crystallographically controlled attachment process. In the ideal case of highly aligned nanocrystallites, OA yields so‐called *mesocrystals*, a term established by Cölfen and co‐workers (see Glossary) [199]. Penn and Banfield broke ground on this growth mode with their prominent report on anatase growth via crystallographically controlled attachment of anatase nanocrystallites [200, 201]. OA has been observed in diverse systems, including synthetic, biological, and geochemical settings [111, 179, 202, 203, 204, 205], e.g., even in bacteria‐generated iron oxyhydroxide [206]. In recent years, OA also grew into a synthetic tool [207] for generating mesostructured minerals [208], functional materials [209], or proteins [210] with high surface area, tailored composition [201, 211, 212], or intricate morphologies [78, 213, 214], such as fivefold twinning structures [215], and fractal, self‐similar features [212, 216, 217]. In situ liquid TEM showed highly directional short‐range particle/particle interactions, with electrostatic and dipolar interactions reaching distances of multiple nanometers [218]. In ferrihydrite formation, primary particles were found to probe various orientational configurations before finally jumping into crystallographically aligned contact over a distance of about one nanometer [219]. The solution/crystal interface has an even more intricate impact on mesocrystal formation, as it can spur nucleation of primary crystallites near the growing mesocrystal. Oxalate‐functionalized hematite (vide supra) does not enhance nucleation in the liquid/crystal interphase; the short distance of about 2 nm to the mesocrystal surface also allows immediate integration of the newly generated nanoparticles [212]. As an interface‐controlled aggregation process, a larger number of factors may affect the process [213], e.g., Ostwald‐like coarsening, surface functionalization, charge, or non‐DLVO interactions.

Recent analyses suggest that OA may be constrained to high initial supersaturations, at which the nucleation rate of nanocrystals is large [220]. Under such conditions, crystals appear in close proximity to each other and have the opportunity to encounter each other, e.g., driven by Brownian motion, and associate before they grow large. OA relies on the premise that an associating particle rotates, driven by Brownian collisions, to an orientation of alignment with the lattice of another crystal within the time that it diffuses over the distance at which it feels the field of that lattice. Crystallites as large 100 nm would require times about 1000‐fold longer to rotate (rotational diffusivity scales as cubed reciprocal size, whereas translational diffusivity is inversely proportional to the size of the diffusing object) than the diffusion time over the nanometer range of the interaction with the lattice of the other crystal.

Alternative mechanisms of nonclassical crystal growth that operate at low and moderate supersaturations have been discovered. Mesoscopic solute‐rich clusters, known to host nonclassical crystal nucleation, were found to land on the surface of growing crystals of proteins and small‐molecule organics [89, 157, 220]. Directed by the periodic field of the underlying crystal lattice, the clusters undergo disorder‐to‐order transformation to structure as crystal layers in complete alignment with the lattice. This process generates a stack of up to 50 layers that spreads along the surface. At slightly elevated supersaturations, layer generation by landing clusters dominates the growth mechanism, and crystal growth is fully nonclassical. Surprisingly, the mechanical and optical properties of the crystals grown nonclassically are identical to those of classically grown crystals, indicating that fast nonclassical growth is an efficient mode to grow technologically important crystals [220].

Dimers and other solute oligomers are other possible agents of nonclassical growth [197]. Understanding the nature of the building block that integrates into a crystal during its growth is critically important. The specific chemical makeup and arrangement of the entity that becomes part of kink sites plays a vital role in both theoretical and applied aspects of crystallization. Models—whether molecular or coarse‐grained—that aim to forecast crystal morphologies and growth dynamics rely on assumptions about this species. Furthermore, the growth unit often becomes the focus of interventions using solvents or additives designed to influence polymorph selection and crystal form. Investigations combining experimental and computational approaches on two organic compounds—olanzapine (OZPN) and etioporphyrin I (EtpI)—have indicated that their crystals form through the incorporation of solute dimers. In the case of OZPN, the prevalence of dimers in the solid phase has led to the hypothesis that these structures preassemble in solution, sequester a substantial portion of the solute, and subsequently nucleate and grow crystals. EtpI, however, arranges in the solid state as parallel arrays of planar monomers with no point symmetry linking them. Despite monomers being the most abundant species in solution for both compounds, OZPN crystal growth at kink sites follows bimolecular kinetics, implying that dimers—though present in lower concentration—act as the effective growth species. The situation is even more complex for EtpI: while its (010) face grows through monomer addition, the (001) face requires dimers as the growth units, further highlighting the disconnect between solution species, incorporation mechanism, and final crystal structure [221, 222, 223, 224].

Association of amorphous precursors can also spur the growth of crystals. Baumgartner et al. showed that magnetite grows by accretion of disordered nanometric primary units without forming an extended amorphous bulk phase [211]. Lupulescu and Rimer showed for zeolite silicalite‐1 that its growth involves both classical and nonclassical trajectories, i.e., the attachment of minimal building molecules and the incorporation of nanometric silica precursors. Cölfen and co‐workers showed for the model system of dl‐glutamic acid a similar multi‐step process, which starts with the formation of clustered solute species and their attachment on crystal surfaces. Upon settlement, these clusters act as 2d nucleators and generate new crystal layers in an otherwise classical, thus monomer‐driven crystallization process [225].

For calcium phosphate, Onuma and Ito observed calcium phosphate cluster formation in the size range of 0.7 to 1 nm in simulated body fluid and also in solution undersaturated against hydroxyapatite and octacalcium phosphate. Via AFM, they concluded that these clusters are the growth units of hydroxyapatite since its crystal growth by step flow features a step height of 0.8 or 1.6 nm [226].

In various biomineralization processes and biomimetic crystallization strategies (see Glossary), such as the PILP process [1], it was shown that amorphous nanoparticles attach and integrate to the growth front of the biomineral. In the case of bivalve nacre, Hovden, Wolf, and co‐workers showed that the first layers of nacre form by a nanoparticle self‐assembly process driven by aggregation of nanoparticles (∼50–80 nm, see Figure 9C). This finding is corroborated by Zhang Xu, who found amorphous immature tablets composed of nanograins [227]. Similar processes have been suggested for other systems, such as sea urchin spines [134, 150, 197, 228]. After integration and additional ripening and dehydration, the crystallization front percolates through the amorphous body and transforms the precursor pseudomorphically into the final crystalline product. The process can be tracked by synchrotron‐based methods such as XANES‐PEEM [109, 110] or PDF [118] mapping. In the case of bivalve nacre or sea urchin tests, these techniques visualize how the crystallization front lags behind the actual mineral growth front. For nacre, XANES‐PEEM shows a proto‐calcitic amorphous precursor that transforms into aragonite (Figure 9A), with scattered intracrystalline islands of still amorphous material. The latter observations suggest that the crystallinity percolates through the biomineral grain by grain [134, 150, 229] (Figures 9B, D), as amorphous islands are in the size range of a single nanogranule (60–120 nm, Figure 9A,B) [110, 229]. The crystal phase and orientation are then imparted to neighboring grains by heterogeneous and homoepitaxial nucleation, reflecting the nanogranular ultrastructure inherited from the prior particle‐mediated deposition process [149, 150]. To highlight the nanoscopic nature of the primary growth‐mediating entities, while not restricting them to explicitly solid particles but also encompassing droplets or solute species, Wolf and Gower put forward the term *colloid assembly and transformation* (CAT, see Glossary) as a generic descriptor for such processes (Figure 10). The pseudomorphic amorphous‐to‐crystalline phase transformation involves shrinkage associated with volume loss, and the resulting transformation strain may generate crystal defects and lattice tilting, as observed in biological and synthetic systems [151, 230, 231]. Protein crystallization represents a distinct case, because crystallization commonly occurs within a condensed protein‐rich phase. Nevertheless, protein systems also seem to yield non‐faceted morphologies, such as spherulitic films [232, 233]. If such morphologies arise from a solid amorphous precursor [232], the corresponding solid‐to‐solid transformation would, strictly speaking, represent a paramorphic rather than a pseudomorphic transformation (see Glossary).

## 4 Untangling Terminology: Discriminating Classical From Nonclassical Processes

### 4.1 The Szilard Postulate: The Major Discriminator

The summary of classical and nonclassical mechanisms of crystal nucleation and growth promotes a central criterion to distinguish both: the Szilard postulate. Thus, systems that comply with the Szilard postulate and nucleate and grow by the association of monomers are viewed as classical. Nucleation enabled by preassembled precursors and growth by the association of solute oligomers, liquid or amorphous particles, and crystallites would be viewed as nonclassical. Importantly, crystals that nucleate nonclassically may still grow classically and vice versa. Furthermore, transitions between classical and nonclassical pathways of growth are common and may be induced by changing supersaturation or the addition of third solution components, an aspect rarely highlighted in nonclassical studies. In this regard, process‐directing polymers, such as PILP‐active polymers, can be particularly efficient third components because they provide several functions in one additive: they may shift association equilibria, stabilize precursor entities, promote assembly, and suppress redissolution or ripening. Related effects, however, might also be achieved by small molecules, ions, solvent components, interfaces, confinement, kinetic process control, or a concerted combination thereof.

The issue of how to transition between classical and nonclassical crystal nucleation is still open and a key question to resolve. Beyond the Szilard postulate as a tell‐tale marker, there are also more subtle cases. Often, a process, e.g., a multi‐step mechanism, is described as nonclassical, while the individual steps may be rationalizable by classical or established concepts. Sometimes it is the convolution of several classical steps folded into one complex process that thereby exceeds the classical scope. One may take two‐step nucleation, an established nonclassical process, as an example: the individual steps may not be surprising, but their entirety escapes classical rationalization. In the following, we will provide guidelines to navigate between the classical and the non‐classical realms of models. Additionally, we highlight some common misconceptions recurring in the literature to provide guidance to circumnavigate such pitfalls.

### 4.2 Defining Nonclassical Nucleation

The centennial CNT carries inherent approximations and implicit assumptions, of which the most prominent ones are:

*The Szilard postulate*: the nucleus grows only by attachment of the smallest possible building units, e.g., individual molecules or ions, and not by larger entities composed of multiple ions/molecules.

*Non‐intermediary processes*: The nucleus formed is structurally identical with the final macroscopic phase. No transient phases, auxiliary processes, or multi‐step processes are envisioned. In classical expansions of CNT, such as the Ostwald step rule, such processes are implicitly assumed to not interfere with the nucleation process besides modulating supersaturation.

*Capillarity approximation*: The nucleus's properties and structure are invariant with respect to nucleus size/curvature; they are identical to the macroscopic phase. Explicitly, the interfacial energy α is approximated by the interfacial energy of the flat macroscopic surface.

*Discreteness*: The nucleus’ phase boundary is smooth, sharp, and discrete; no interphase affects phase separation processes.

*Sphericality*: The nucleus adopts a spherical or, in the case of crystals, isometric shape.

*Binary energy balance*: The free energy balance is sufficiently described by considering surface and volume energy. No other contributions are relevant, e.g., entropy or short/long‐range attractive forces. Since both contributions scale with size, the nucleus size becomes a critical descriptor.

*Phase domain isolation*: CNT considers the fate of isolated nuclei; any interaction (e.g., coalescence or aggregation) or competition between those is not considered.

About half a century before CNT, Gibbs already expected that globulae (his term for nuclei) feature a chemical composition and interfacial tension distinct from the mature phase [16], thus already objecting to both the capillarity approximation and the absence of transitory stages. Still, these approximations were later introduced, mainly to render the model conceptually and mathematically manageable. There has always been awareness of issues arising from those approximations, already in the early days of CNT, e.g., as uttered by Neumann and Döring [234, 235]. Later years brought many further examples of incorrect or even unphysical predictions of CNT, some with nucleation rates too low by a factor of 10^−30^ −  10^−50^ [236, 237, 238, 239, 240, 241]. Thus, it is unsurprising that CNT has conceptual limits and falls short in explaining experimental findings. These classical approximations echo the original model system, for which most assumptions above are reasonable.

***Nonclassical nucleation*** typically breaches one or more of CNT's assumptions listed above. However, only sufficiently severe breaches that lead to CNT's conceptual breakdown are considered hallmarks of nonclassicality. Subordinate breaches only reveal more shortcomings of the classical framework. For instance, e.g., non‐sphericality is mostly deemed a subordinate breach, since already for crystal nucleation the sphericality assumption collapses immediately. Similarly, for globular proteins with aeolotropic (orientation‐dependent) interactions [198], the sphericality assumption becomes invalid; e.g., quasi‐planar nuclei [242]. Typical breaches that sufficiently legitimate nonclassical nucleation schemes are:

i. intermediary processes, e.g., two‐ and multi‐step nucleation.
ii. process‐decisive energy contributions beyond the classical binary energy balance that allow for nucleation triggers uncorrelated with the nucleus size.
iii. nucleus growth by larger entities (e.g., ion associates or supra‐molecular/‐ionic species) or particle interaction (e.g., P2 entities in gypsum formation).

### 4.3 Defining Nonclassical Crystal Growth

The above “canonical” growth concepts are coherent in their assumption that these processes are driven by attachment and incorporation of individual, isolated ions or molecules and growth proceeds without incurring structural changes, as reviewed in Section 2. In contrast, Section 3 provided several instances in which this assumption is violated, evidencing crystal growth driven by diverse colloidal, nanoscopic entities. Note that once a nucleation germ has transitioned into a growing particle of the new phase, the system has essentially left the scope of CNT and entered the growth stage.

***Nonclassical crystallization*** occurs when growth is fueled by larger entities comprising more than one minimal growth unit (i.e., ions or molecules), e.g., supra‐molecular or supra‐ionic species (e.g., ion associates, coordination entities, or mesoscopic solute‐rich clusters) or colloidal species (e.g., nanodroplets or nanocrystallites) [243]. These larger growth units may leave a structural imprint on the forming solid, e.g., mesocrystallinity [208] or nanogranularity [134, 228]. However, the presence or absence of a mesocrystalline stage is not a sufficient condition for a nonclassical crystal growth process [244]. Moreover, a classical‐looking final morphology does not exclude a non‐Szilard pathway, because later redissolution, ripening, or classical transformation may erase earlier nonclassical signatures.

This definition reflects the common consensus for *crystallization by particle attachment* (CPA) via nanocrystals [219], amorphous nanoparticles [110, 149], or liquid droplets [111, 126, 245] as documented by De Yoreo et al. in a comprehensive overview. Note, however, that while the term “particle” in CPA may broadly include liquid droplets and even complex solutes, the more general term “colloid”, as used in CAT, may be more fitting in this context [141, 142]. The predominant thermodynamic driving force for such aggregational attachment processes is reduction in surface free energy. Usually, the systems are kinetically geared towards nanoparticle formation due to burst nucleation [71] (e.g., under LaMer processes) or nanoparticle‐stabilizing capping agents [207], to leverage colloid/colloid interactions (e.g., for OA). PILP‐related processes can be seen as nonclassical in their nucleation and growth behavior: they feature a two‐ or multistep nucleation mechanism, and the initial amorphous body grows not by smallest‐possible units but larger entities. There is still much to be learned about how additives such as polymers and proteins modulate these nonclassical pathways.

### 4.4 Circumnavigating Common Pitfalls

The mere and detached occurrence of an amorphous phase is not a valid sign of a nonclassical process, neither concerning nucleation nor crystal growth. Ostwald's step rule is fully in line with classical concepts; it highlights that amorphous phases in early stages of polymorphic systems are classically expected. It is only a nonclassical system if the amorphous phase impacts subsequent nucleation and growth processes beyond the classical canon. Moreover, the mere occurrence of a liquid phase, e.g., during an Ostwald step process, is not indicative of a nonclassical or spinodal process. Like solids, liquid phases usually form via nucleation; in fact, CNT explicitly treats the nucleation of a liquid in its supersaturated vapor. Ostwald expected a liquid phase as the first phase in an Ostwald step process due to the low interfacial energy with the parental solution [73].

Spinodal demixing is common for a specific class of phase‐separating systems in which it is experimentally feasible to achieve a deep and spatially extended *T*‐ or *p*‐quench of a homogeneous system into the instability regime: here, reduced diffusion becomes decisive. Spinodal demixing is thus reported for systems such as glasses, alloys, or soft‐matter systems, all of which feature low diffusivity. Solutions of small solutes feature high diffusion rates; hence, homogeneous quench into instability becomes challenging, and burst nucleation is prone to take over, especially when supersaturation is increased by gradually increasing concentration (see Section 2). Although the absence of an activation barrier is characteristic of spinodal demixing, it is not unequivocal and sufficient evidence for the occurrence of spinodal processes, especially if only computationally determined. Aggregation processes, such as gelation, may also show the feature of a lacking activation barrier to phase separation, while lacking other predicted key features of spinodal demixing [246, 247]. To paraphrase PNC formation as localized spinodal decomposition contradicts their solute state; only if they represent indeed a phase would such a description be appropriate.

A concentration fluctuation is not a phase; in contrast to the latter, fluctuations are mere local solute enrichments that are not persistent in time or delineated by a sufficiently sharp phase boundary from a surrounding matrix phase. Thus, a liquid‐condensed phase, e.g., a delineated droplet in a parent solution, must be terminologically discriminated from solute clustering. Solute clusters, e.g., ion pairs and associates, are still part of the solution, whereas a phase boundary defines a liquid‐condensed phase. Ion pairs and larger ion associates are distinct chemical species and may even be considered molecular entities since contact ion pairs can exhibit altered chemical properties and reactivity [248, 249]. Various criteria have been proposed to define ion pairs and associates, including lifetimes exceeding the correlation time in Brownian motion, binding energies exceeding *k_B_T*, or interionic cut‐off distances. Discrimination between ion pairs/associates and coordination species can become artificial or—as put prominently by Marcus and Hefter [249]—both may be regarded as “essentially indistinguishable or just slightly different aspects of the same phenomenon”. Accordingly, close‐contact ion pairs and larger ion associates may be viewed as coordination‐type species, while acknowledging that terminology differs between electrolyte theory and coordination chemistry [249].

The term cluster has diverse meanings across disciplines. In CNT, the original terms nucleus or embryo are today often replaced by terms such as prenucleation or post‐nucleation cluster. In physics, a cluster usually denotes more of an aggregate, with the inter‐constituent attachment ranging from loose to tight. In bioinorganic and coordination chemistry, by contrast, the IUPAC definition describes a cluster as “a number of metal centres grouped close together” [3], which may involve interactions through bridging ligands, i.e., a polynuclear coordination species [3]. The term prenucleation cluster, therefore, remains intrinsically ambivalent, and its use varies across the literature [169, 250]. It may carry an “ancestral” interpretation as a proto‐nucleation cluster, i.e., a premature nucleus that requires a trigger or a process to ripen into an active nucleus. Viewed from CNT, a prenucleation cluster may imply cluster formation under undersaturated conditions. Alternatively, in a purely chronological sense, a prenucleation cluster could refer to a supra‐ionic species or cluster that exists before nucleation. Moreover, the coordination‐chemical term cluster refers to polynuclear coordination species, whereas monocenter entities such as [Ca(HPO_4_)_3_]^4–^ (vide supra) would be excluded, although such species may contribute to nonclassical nucleation pathways. To avoid these semantic ambiguities, alternative expressions such as *prenucleation species* or *supra‐ionic species* have been introduced. The former is a purely temporal term encompassing all solute species present before nucleation, whereas the latter more specifically denotes associated species beyond single ions (see Glossary) [184, 243, 251, 252]. Evidencing the involvement of a cluster species in phase separation processes is thus essential for properly assessing nonclassicality of a mineral system.

Note also that mesocrystal formation via amorphous particle aggregation (e.g., CAT, CPA) cannot be seen as a subset of or described as oriented attachment since isotropic materials such as amorphous mineral precursors cannot undergo directionally‐preferred accretion. Also, one should clearly distinguish between nonclassical nucleation and nonclassical growth mechanisms; the one does not necessarily follow the other.

### 4.5 Toward a Unifying Concept Across Organo‐Polymer and Inorganic Systems

Neglecting solution chemistry and colloidal interactions are the major shortcomings of the classical theories in all classes of systems. Organic systems are drastically different from inorganic ionic crystals. Interactions are dominated by weak, non‐directional van der Waals forces. These interactions enable the formation of mesoscopic solute‐rich clusters in many organic systems. The mesoscopic clusters provide a concentrated environment for the formation of crystal nuclei, in which the surface free energy of the crystal‐cluster phase interface is lower by about 10‐fold and the barrier confronting crystal nucleation is lower by many decades. As a result, crystallization hosted or mediated by the mesoscopic clusters is substantially faster than classical nucleation by molecular assembly in the solution, and the nonclassical nucleation pathway is strongly preferred.

**FIGURE 12:**
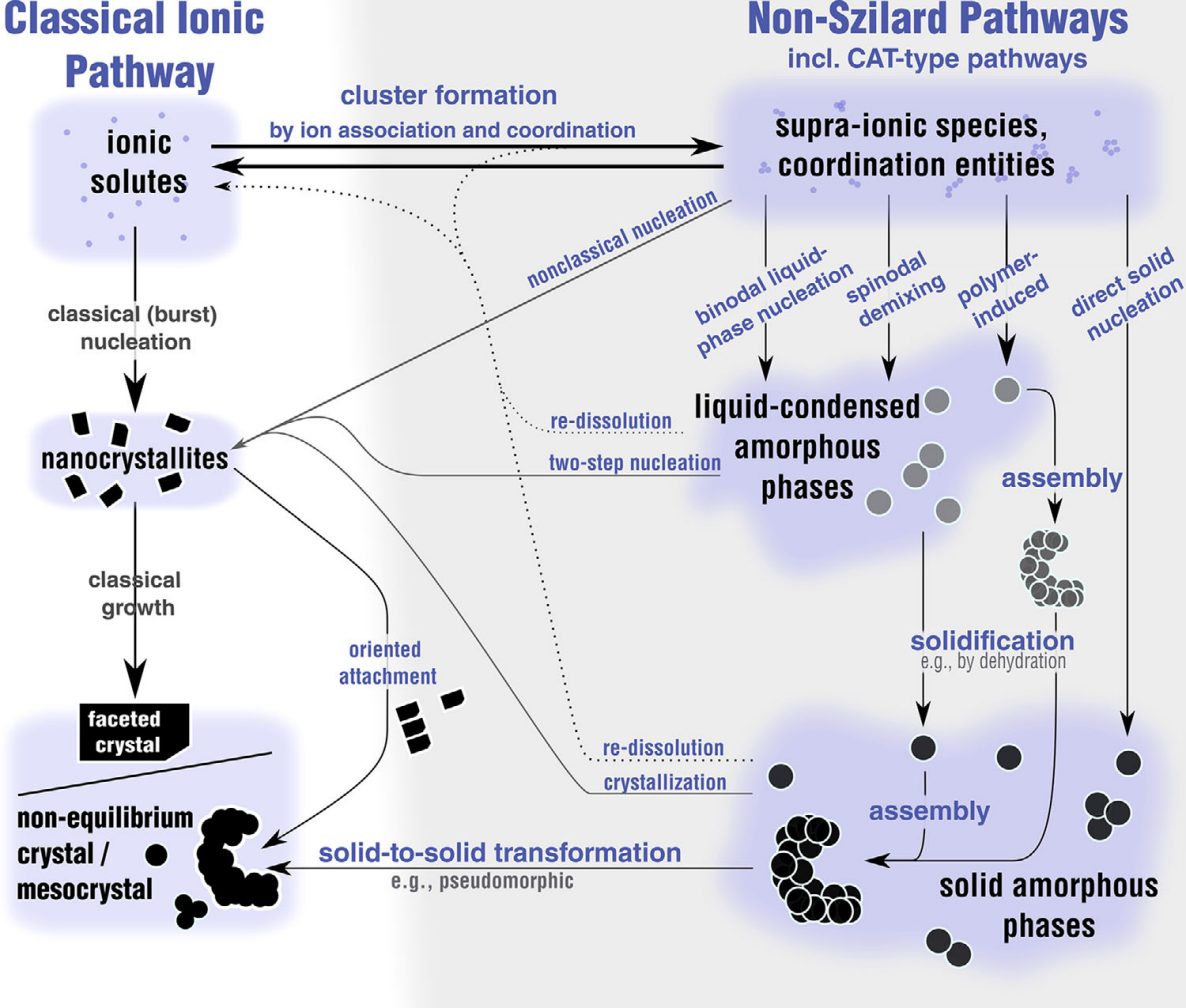
Schematic representation of putative mechanistic cross‐overs in ionic inorganic systems, in which classical and nonclassical/non‐Szilard pathways may coexist. Ion association and coordination equilibria, including ion pairing, can generate supra‐ionic species or coordination entities, such as PNCs, ion associates, or coordination networks. These entities may undergo phase‐separation behavior reminiscent of soft‐matter systems, including binodal liquid‐phase nucleation, spinodal demixing, polymer‐induced condensation, or direct solid nucleation. Depending on association and coordination equilibria, their kinetics, and the reaction conditions, the classical ionic pathway or non‐Szilard pathways may dominate. Process‐directing additives, including cluster‐stabilizing additives or polymers, provide an additional lever for reaction engineering by shifting association/coordination equilibria or stabilizing condensed precursor states. Oriented attachment is shown as a distinct non‐Szilard route that proceeds via nanocrystallites, whereas CAT‐type pathways involve the assembly of amorphous precursor bodies and their subsequent transformation. White borders indicate phase boundaries.

How can seemingly simple ionic mineral systems exhibit phase‐separation behavior reminiscent of proteins or organic molecules? Prenucleation clusters and related species, whether mononuclear or cluster‐like, constitute molecular‐scale colloidal entities. The transition from a solute species to a colloidal entity is gradual, with increasing size and association progressively shifting its behavior from molecular speciation toward particle‐like interactions. Cluster formation largely screens and internally counterbalances the strong long‐range Coulomb interactions of individual ions. As a consequence, the reduced electrostatic repulsion between these colloidal entities allows ever‐present short‐range attractive interactions, such as van der Waals and hydrogen‐bonding interactions, to become decisive for inter‐species interactions. Such supra‐ionic species can thus display interaction characteristics akin to those of molecular or colloidal systems, giving rise to behavior that is atypical for ionic systems but common in soft matter, including assembly and liquid/liquid phase separation. While such phase separation is regularly observed in proteinaceous systems, it is regarded as extraordinary in mineral systems. However, once supra‐ionic species are recognized as relevant actors in phase‐separation pathways, otherwise surprising observations become plausible, as illustrated in Figure 12.

Suitable reaction conditions may amplify cluster prevalence and, thereby, enhance the impact of weak interactions on phase‐separation behavior. Biomineralizing systems appear to have mastered a superb level of process control by using tailored proteins and other modifiers, such as Mg ions, to exploit and regulate these previously underrecognized nonclassical species and thereby pull an inorganic ionic system into the soft‐matter realm, with self‐assembly mechanisms operating on length scales that can be precisely controlled by biology. The emerging structural complexity and exceptional material performance of biominerals demonstrate the synthetic potential that we can unlock by further fathoming nonclassical nucleation and crystallization.

A key lesson learned from colloid science is that the starting conditions do matter and co‐determine structure formation. Hence, phase separation via molecular pathways will also depend strongly on the starting position in the phase diagram, and kinetic factors of the phase transition play a particularly important role [246]. Currently, inorganic systems are often perceived as following only one major mechanism, independent of varying reaction conditions (e.g., reaction procedures/kinetics, salt and spectator ions). Future research should therefore also pay close attention to these “soft” parameters and map out their impact on the course and nonclassicality/classicality of the phase separation processes.

## 5 Outlook

Fathoming the mechanisms of nucleation and crystallization is, metaphorically, a fractal undertaking. Although our view is often limited by the technical difficulty of capturing intrinsically stochastic processes in their entirety and is therefore frequently based on partial snapshots, increasing spatiotemporal resolution reveals ever more ramified and convoluted process details, such that overly generalized models increasingly reach their limits. At the same time, identifying commonalities across dissimilar systems and disparities among similar ones will provide key cornerstones for building the still‐missing unifying concept of solid‐state genesis in solution.

While exploring this still unchartered territory, a descriptive terminology arises, often ad hoc, and which develops over time. The clear‐cut distinction between classical and nonclassical processes implies an antagonistic situation, in which only one of the two prevails. This is a false impression. We should perceive nonclassical and classical theories as complementary and synergistic, highlighting different sides or reaction pathways of a multifaceted phenomenon; each with its own scope — similar to Newtonian and Einsteinian physics.

Recent years have seen intense discussion concerning novel terminology, such as mesocrystals or prenucleation clusters. Such semantic discussions demarcate paradigmatic shifts, and this kind of emerging opposition to new concepts is inherent to scientific revolutions—as highlighted by Kuhn [253]. Haüy may serve us as an example: he introduced the groundbreaking idea of the unit cell using the term “integrant molecule”, a terminological mistake that is unfitting and misleading when judged from today's point of view but a groundbreaking scientific concept. This account of classical and nonclassical concepts is our contribution to catalyzing the conceptual, terminological transition and helping researchers navigate the field during this transitory stage.

## 6 Glossary

*This glossary aggregates established definitions for key terms with a focus on those especially critical or debated terms. The definitions are based on international bodies or accepted contributions in the field*.

***Association in solution***. Attraction, which may be electrostatic, van der Waals, hydrophobic, or other, can allow solutes to form *associates*. Oppositely charged solutes can form *ion pairs* and *ion associates* in solution; see there.

***Biominerals vs. Biomaterials vs Biomimetic Materials***. *Biomineralization* is mineral formation driven by living organisms. This includes biosynthesis of structural components, such as mineralized tissues like bone, and by‐products due to metabolic activity. Although biominerals do not strictly comply with current international mineralogical association (IMA) definitions, they are commonly accepted as a subset of mineral materials [254]. Minerals synthesized in vitro, without the action of living organisms, *are not* biominerals — they are products of bio‐inspired or biomimetic mineralization routes. Biominerals and biomimetic/‐inspired materials are also often confused with *biomaterials*, which in its primary definition [255] denotes a (mostly synthetic) material designed to treat, augment, or replace (parts of) an organ or body functions (e.g., bone and teeth replacements).

**Colloidal Assembly and Transformation (CAT)**: a term introduced by Wolf and Gower [141] as an overarching concept for mineral/crystal growth via transient precursors. It emphasizes the nanoscopic and colloidal nature (∼1 nm to ∼1 µm) of the primary growth‐mediating entities. In the CAT framework, nanoscale colloidal entities first assemble—potentially under the influence of the surrounding compartment, matrix, or substrate—and subsequently transform into a thermodynamically more stable phase. CAT extends the PILP concept beyond some of its implicit limitations by avoiding a strict assignment of the precursor to either a purely liquid or fully solidified state, making it applicable to a broader range of systems and process trajectories. For instance, the CAT pathway accommodates precursor bodies that may be liquid‐like, viscoelastic, gel‐like, partially coalesced, or progressively densifying, and in which crystallization may proceed along different stages of this evolving precursor state. By coupling colloid assembly to a shape‐preserving para‐ or pseudomorphic transformation, CAT can give rise to non‐equilibrium crystal morphologies [1, 111, 133, 229]. Examples include diverse biominerals (Figure 9) and PILP‐like processes (Figure 10). CAT and OA represent distinct mesocrystallization pathways that differ in the sequence of structural evolution: in CAT, crystallinity arises after assembly, whereas in OA it is already present at the time of attachment. See also refs. [141, 142].

***Crystals*** are defined by their properties in direct or reciprocal space, a dichotomous definition that is an outcome of Shechtman's discovery of quasicrystals lacking translational order [256]. According to IUCr [60]: “A solid is a crystal if its atoms, ions and/or molecules form, on average, a long‐range ordered arrangement” in direct space. In reciprocal space, a crystal “has essentially a sharp diffraction pattern;” the term “essentially” requires that most of the scattering intensity contributes to relatively sharp Bragg peaks. This definition becomes ambiguous for very small nanoparticles (e.g., ∼2 nm), since no clear cut‐off exists for long‐range order or peak sharpness.

**Ion pairs and associates**. In *loose ion pairs*, the associating ions are separated by (solvent) molecules; in a solvent‐separated ion pair, both ions’ solvation remains, while in a solvent‐shared ion pair a single solvent layer solvates both ions. *Tight* or *contact ion pairs* are in direct vicinity with no solvent molecules in between the ions. Larger loose or tight associates may form, giving rise to supra‐ionic species, clusters, or aggregates. Contact ion associates are “essentially indistinguishable” from coordination compounds [248, 249].

***Mesocrystals***: Mesoscopically ordered crystals [78, 199], composed of crystallographically co‐aligned nanocrystallites [214]. The resulting mosaic crystal [257] exhibits low mosaicity —sometimes near coherence—so that a mesocrystal may behave like a single crystal in certain aspects (e.g., scattering) but as a particle assembly in other regards (e.g., surface). Cölfen, Sturm, and co‐workers defined a mesocrystal as “a nanostructured material with a defined long‐range order on the atomic scale, which can be inferred from the existence of an essentially sharp wide‐angle diffraction pattern (with sharp Bragg peaks) together with clear evidence that the material consists of individual nanoparticle building units” [208, 216]. Originally linked solely to oriented attachment, mesocrystals are now recognized to form via additional pathways such as CAT or CPA in both biominerals and synthetic systems (Figures 9 and 10, see also Glossary entry CAT).

Today, the term denotes only a material's structural organization, as verified by detailed characterization [244]. Mesocrystals can ripen into single crystals, losing their mesocrystalline identity [216].

***Minerals***: chemical compounds or elements, typically crystalline, formed by geological processes, as defined by the international mineralogical association (ima) [258]. They must be solid (with mercury as the sole liquid exception), stable, and chemically well‐defined. The formal definition (fulfillment required for official mineral naming) excludes biominerals and purely synthetic analogues, while amorphous solids qualify if composition is well defined. In interdisciplinary contexts, usage of the term mineral has broadened to encompass “an element or compound, amorphous or crystalline, formed through biogeochemical processes,” thereby including biominerals as part of the mineral kingdom [254].

***Prenucleation clusters*** (PNCs) are nanosized ion associates proposed to exist as an ergodically stable solute population prior to nucleation. They are expected to form even under undersaturated conditions, lack a phase boundary, and have been discussed as mechanistically relevant species in nonclassical nucleation pathways. See also refs. [169, 178] for a more elaborated definition.

***Prenucleation species***: all solute species present in solution prior to nucleation, including ions, molecules, ion pairs, and larger associated species or clusters. The term is purely temporal and does not imply any mechanistic role in (nonclassical) nucleation; it therefore encompasses both single ions and prenucleation clusters (PNCs). PNCs are thus not synonymous with prenucleation species, but represent a specific subset thereof.

***Pseudomorphic transformations*** preserve external form despite chemical and/or structural change, whereas *paramorphic transformations* preserve form while only the crystal structure changes, but composition remains unchanged.

***Supra‐ionic species***: an umbrella term for prenucleation species larger than single ions. The term encompasses inorganic(‐dominated) solute species that often include additional molecular components (e.g., coordinated water). The term includes PNCs but remains agnostic regarding any mechanistic role of such species in subsequent phase separation. Structurally, some of these supra‐ionic species may also qualify as *coordination polymers* [3], i.e., coordination compounds with repeating coordination entities extending in one, two, or three dimensions, whereas *coordination networks* [3] constitute their cross‐linked or intrinsically 2D/3D subset.

## Funding

SEW is a Heisenberg Fellow of the Deutsche Forschungsgemeinschaft (DFG, Grant No. 501391584) and acknowledges further financial support from the DFG (Grant No. 501387034 and 554953245). PGV acknowledges the support of the National Science Foundation (Grant DMR‐2128121) and the Welch Foundation (Grant E‐2170 and the Welch Center for Advanced Bioactive Materials Crystallization, Award V‐E‐0001).

## Conflicts of Interest

The authors declare no conflict of interest.

## Endnotes

1 Original German: “dass beim Verlassen irgend eines Zustandes und dem Übergang in einen stabileren nicht der unter den vorhandenen Verhältnissen stabilste aufgesucht wird, sondern der nächstliegende.” English translation by S.E.W.
